# The National Food Consumption Survey IV SCAI: Nutrient Intakes and Related Dietary Sources in Italy

**DOI:** 10.3390/nu18010088

**Published:** 2025-12-27

**Authors:** Cinzia Le Donne, Marika Ferrari, Lorenza Mistura, Laura D’Addezio, Francisco Javier Comendador Azcarraga, Deborah Martone, Raffaela Piccinelli, Stefania Sette, Giovina Catasta, Aida Turrini

**Affiliations:** 1 Council for Agricultural Research and Economics (CREA), Research Centre for Food and Nutrition, 00178 Rome, Italy; marika.ferrari@crea.gov.it (M.F.); lorenza.mistura@crea.gov.it (L.M.); laura.daddezio@crea.gov.it (L.D.); fjavier.comendador@crea.gov.it (F.J.C.A.); deborah.martone@crea.gov.it (D.M.); raffaela.piccinelli@crea.gov.it (R.P.); stefania.sette@crea.gov.it (S.S.); giovina.catasta@crea.gov.it (G.C.); 2 Independent Researcher, 58054 Scansano, Italy; aida.turrini@gmail.com

**Keywords:** usual dietary intake, nutrient adequacy, food sources, Italian population, national survey

## Abstract

**Background/Objectives**: The Fourth Italian National Food Consumption Survey (IV SCAI 2017–2020) provides updated and comprehensive data on the dietary habits of the Italian population. The study aimed to assess nutrient intakes and their main food sources among individuals aged 3 months to 74 years and to evaluate the adequacy of intakes against the Italian dietary reference values (DRVs). **Methods**: A nationally representative sample of 1969 participants were surveyed using two non-consecutive food diaries (ages 3 months–9 years) and 24 h recalls (ages 10–74 years) in accordance with the European Food Safety Authority’s EU Menu guideline. The multiple source method was used to estimate the usual intakes accounted for intra-individual variability. Nutrient adequacy was assessed against age- and sex-specific DRVs, and the main food sources of macro- and micronutrients were identified. **Results**: Energy intake was below DRVs for adults, particularly women, while protein intake exceeded recommendations across all ages, mainly from animal sources (67% of total). Total fat (38%En) and saturated fat (12%En) exceeded the recommendations, whereas carbohydrates (45%En) and dietary fibre were suboptimal. Vitamin D and calcium intake were markedly below DRVs for all age groups; iron inadequacy was prevalent among females. The main energy sources were cereals (39%), milk and dairy (15%), oils and fats (13%), and meat (10%). Vegetables and fruits were leading contributors to vitamins A and C, while meat, fish, and dairy provided vitamin B12 and D. **Conclusions**: The Italian diet remains cereal-based but shows nutritional imbalances: notably, excessive protein and fat intake and widespread deficiencies in vitamin D, calcium, iron, and fibre. These findings underline the need for targeted nutrition policies to realign dietary patterns with the national recommendations.

## 1. Introduction

National food consumption data represent an essential evidence base for the development of coherent, effective, and equitable food policy programmes [1,2]. Dietary surveillance systems that collect such data allow for the assessment of nutrient adequacy and of the food sources, providing crucial insights into the quality and diversity of diet. These aspects are important for guiding interventions in both the public health and agricultural policy domains [3].

Within the framework of the European Food and Nutrition Action Plan, the WHO explicitly encourages member states to strengthen local surveillance programmes and conduct nationwide nutrition surveys, thereby highlighting the need for valid, representative, and harmonised data [4,5].

From a public health perspective, nutrition is a key determinant of health across the entire life course. Balanced dietary patterns provide essential nutrients that sustain physiological homeostasis and, consequently, shape health outcomes [6]. Conversely, unhealthy or suboptimal diets are among the leading global risk factors for mortality and morbidity. They substantially contribute to the burden of non-communicable diseases (NCDs), including cardiovascular disease, obesity, type 2 diabetes, and several types of cancer [7,8]. Likewise, inadequate diets can result in stunting, wasting, underweight, developmental delays, higher susceptibility to infections, and an overall increased risk of adverse health outcomes [9].

Nutrients are conventionally classified into macronutrients and micronutrients, with each category fulfilling distinct but complementary roles in metabolism and physiology [10]. The identification of nutrient inadequacies at population levels (e.g., insufficient intake of fibre, iron, or vitamin D), as well as excessive consumption of nutrients of concern (e.g., sodium, free sugars, saturated fats), enables the design of targeted interventions aimed at shifting consumer choices towards healthier options [11]. In this regard, dietary data that are disaggregated by age, gender, socioeconomic status, and region enable the identification of vulnerable subgroups at higher risk of malnutrition, whether in the form of undernutrition or overnutrition. Such granularity supports the development of equity-focused policies, ensuring that interventions are tailored to the specific needs of different communities [12]. Previously, the third Italian National Food Consumption Survey (INRAN-SCAI 2005-06) highlighted key nutritional imbalances in the observed dietary patterns in Italy, including excessive saturated fat and sugar intake and insufficient fibre and micronutrients [13]. These findings supported the Italian Dietary Guidelines [14] in providing targeted recommendations—such as increasing fruit, vegetables, whole grain foods, and legumes consumption, and reducing foods that are high in fats, sugars, and salt—to address the specific dietary needs of the Italian population groups.

Moreover, the nutrient intake and dietary source data are also important for agricultural and food production planning. Understanding dietary intake aligns agricultural output with nutritional needs, ensuring the availability of sufficient quantities of diverse, nutrient-rich foods [15]. In this regard, a simulation study demonstrated that integrating dietary data with agricultural and environmental projections can help design strategies to mitigate nutrient inadequacies [16]. This Italian case study on projected iron intake—evaluating different scenarios of wheat and rice biofortification and whole-grain food consumption—highlights the potential of coordinated agricultural and dietary interventions to counteract the adverse nutritional effects of climate change and support the achievement of adequate micronutrient intakes [16].

Beyond nutritional adequacy, the composition of diets is a central determinant of food system sustainability. Diets rich in plant-based foods, minimally processed items, and locally sourced products can reduce environmental footprints—lowering greenhouse gas emissions, land use, and water consumption—while simultaneously contributing to health promotion [17,18]. Conversely, high consumption of animal-based, highly processed, or resource-intensive foods can exacerbate environmental degradation and compromise long-term food security. Recent evidence indicates that healthier dietary patterns are generally associated with lower environmental impacts and greater adherence to sustainable lifestyles [19,20,21]. The Italian Mediterranean dietary model adapted to the EAT-Lancet reference diet (EAT-IT) has likewise been demonstrated to reduce carbon and water footprints while maintaining nutritional adequacy [22]. Integrating dietary patterns into sustainability strategies helps ensure that food policies promote not only nutrition but also environmental stewardship, social equity, and economic resilience [23].

The aim of this study was to provide a comprehensive evaluation of nutrient intake patterns that emerged from the Fourth Italian National Food Consumption Survey (IV SCAI, 2017–2020) in terms of nutritional adequacy compared to the Italian dietary reference values (DRVs) [24] for different age groups. Furthermore, the main dietary sources of the nutrients of public health concern are identified. These results provide updated evidence to inform nutrition and public health policies, guide targeted interventions, and support agricultural and food-system planning to meet the population’s nutritional needs.

## 2. Materials and Methods

A detailed description of the study design, participation rate, and survey protocol can be found elsewhere [25,26,27,28,29]. The main features are summarised below.

### 2.1. Study Population and Data Collection

The IV SCAI national consumption survey was carried out by the CREA Research Centre for Food and Nutrition between June 2017 and January 2020, in compliance with the principles, established protocols, and guidelines of the EU Menu project [30]. A total of 1969 individuals (48% male) living in Italy, ranging in age from three months to 74 years, and stratified by sex and geographical area (northwest, northeast, centre, south, and islands), participated in the food survey. The age groups involved were infants (3–11 months); toddlers (1–2 years); children (3–9 years); adolescents (10–17 years); adults (18–64 years); and the elderly (65–74 years). Dietary assessment was based on two non-consecutive food diaries for children aged 3 months to 9 years and on two non-consecutive 24 h dietary recalls for participants aged 10–74 years, separated by at least 15 days. Consumption of foods, beverages, and food supplements was collected by trained fieldworkers (paediatricians, biologists, nutritionists, and dietitians), and the relative quantity was estimated by using two specially developed picture books for children and adults [31,32,33,34,35,36] and household measures (e.g., glasses and cups) or expressed directly in weight/volume or standard units (e.g., commercial food portions). Anthropometric data (body weight and length/height) were measured by trained interviewers, using standardised procedures according to WHO recommendations [37]. A questionnaire was also administered to collect information on the socio-demographic characteristics of the participants or their parents/caretakers (in the case of the child population).

The study was conducted in accordance with the Declaration of Helsinki. Written informed consent was obtained from all enrolled subjects before proceeding with study interviews. For children, information was provided to parents, guardians, or other legal representatives. The study was approved by the Ethics Committee of ‘Lazio 2’ (Rome, Italy, protocol code 106872/2016, 19 October 2016) and by the Ethics Committee of the National Institute of Health (Rome, Italy, protocol code AOO-ISS 0028469, 24 September 2018).

### 2.2. Data Entry, Food, and Nutrient Databases

Dietary information was entered by trained interviewers using the continuously updated software ‘FoodSoft 1.0’. This software and all associated databases, including those for food nutrient composition, food nomenclatures, and portion size data, were developed by the CREA Research Centre for Food and Nutrition [38,39] for use in nutritional and epidemiological studies [40,41,42].

The current, complete version [43] includes two data entry modules: the 24 h recall and the food diary, as well as “data management” features. The 24 h recall module permits data entry using the multiple-pass method, in accordance with EU Menu guidelines [30].

The software has been carefully designed to guide users through each stage of the data fill-in process. Its output provides a comprehensive description of the foods consumed, including the amount in grams (expressed as raw net amount); water and overall energy intake (En); macronutrients (protein, total fat, and fatty-acid classes such as saturated (SFA), monounsaturated (MUFA), and polyunsaturated (PUFA) fatty acids); available starch and soluble carbohydrates (CHO); dietary fibre; alcohol; and cholesterol. The system also reports a wide range of micronutrients, including minerals—such as calcium (Ca), phosphorus (P), magnesium (Mg), potassium (K), iron (Fe), and zinc (Zn)—and vitamins, including vitamin C, thiamine, riboflavin, niacin, vitamins B6, B12, D, E, and K, retinol, β-carotene, and vitamin A (expressed as retinol equivalents, REs), as well as dietary folate equivalents (DFE), natural folate, and folic acid (from fortified foods and supplements) [43]. The nutrient database has been thoroughly and comprehensively completed, encompassing all nutrients present in each food item. The food list was largely composed of foods consumed in the previous national survey, ‘INRAN-SCAI 2005-06’ [40], which, to date, includes 3245 food items (2001 main foods, 1244 synonyms), 1523 recipes (1267 main dishes and 256 synonyms), and 439 dietary supplements. A lexicon of synonyms for foods and recipes has been developed to facilitate accurate identification during data entry, allowing for different popular and regional names. The classification of all foods, recipes, and food supplements was conducted in accordance with the FoodEx2 system [44,45,46]. This system was utilised for the purpose of incorporating the aforementioned items into the EFSA Comprehensive European Food Consumption Database [47]. Nevertheless, for the present study, the categorisation employed in the consumption database of the Italian population [28,40], following appropriate revision, was essentially considered (Appendix B).

### 2.3. Data Analysis

All analyses were conducted by using sample weights representing the Italian population in 2019, stratified by age group. Usual dietary intake was estimated using the multiple source method (MSM) [48], which adjusts for both inter- and intra-individual variability. MSM is a web-based platform developed with open-source components, written in Perl using the Catalyst framework, and it relies on R as the statistical engine through the Statistics: R package (R version 4.5.2). The tool is accessible at https://nugo.dife.de/msm/, (Version 1.0.2e) (accessed on 1 March 2025).

The distribution of participants’ usual energy and macro- and micronutrient intake was described by using means and standard deviations, medians, and the 5th, 10th, 25th, 75th, 90th, and 95th percentiles by age group and sex. Nutrient adequacy was assessed by comparing usual nutrient intakes with age- and sex-specific reference values from the Italian dietary reference values (DRVs) [24]. The DRVs include several nutrient-based indicators with different purposes, providing the average requirements (AR), as well as recommended population intakes (PRI) or adequate intakes (AI). The most appropriate indicator available for each nutrient was applied. The percentage of contribution of major food groups to the total energy and nutrient intakes was calculated to identify the primary dietary sources of each nutrient.

Infants, school-aged children, and adolescents were classified according to age- and sex-specific BMI z-score cut-offs proposed by the WHO for ages 0–5 years and 5–19 years [49,50,51], using WHO AnthroPlus software [52]. For adults and elderly people, categories followed the WHO criteria [53] (underweight: BMI < 18.5; normal: 18.5 ≤ BMI < 25; overweight: 25 ≤ BMI < 30; obese: BMI ≥ 30). Statistical analysis was performed by using SAS software, version 9.4 (SAS Institute, Inc.; Cary, NC, USA).

## 3. Results

The IV SCAI sample included 1969 individuals (954 males and 1015 females) between the ages of 3 months and 74 years, who completed two-day food records or 24 h recall interviews. The main characteristics of the sample in terms of height, weight, and body mass index (BMI) by all IV SCAI age groups and sex and other information have been described previously in Mistura et al. [28] and have also been reported in Table 1. In brief, adult males had a significantly higher BMI and prevalence of being overweight compared to females. Overall, 11.8% of the population was obese and the highest prevalence of obesity and overweight was found in the south and the islands (5.7% and 9.4%, respectively), followed by the centre (2.1% and 4.5%).

This paper presents an analysis of the intake of selected health-relevant nutrients and their main dietary sources. Results are disaggregated by sex and age group (3–9 years, 10–17 years, 18–64 years, and 65–74 years). The Supplementary Materials provide comprehensive data on all nutrients contained in the IV SCAI database, including those for younger age groups of 3–11 months and 1–2 years (Tables S1–S7—Nutrient intakes), as well as their corresponding food sources (Tables S8–S14—Dietary sources).

### 3.1. Nutrient Intake and Adequacy

Usual intake of energy, macro-, and micronutrients (including fortified foods but excluding supplements) by sex and total sample is presented in Table 2. The data provide an overview of mean intake values, individual variability (standard deviation), and intake distribution across percentiles (P5, P25, P50, P75, P95).

The mean energy intake was 1667 kcal/day (6.97 MJ/day) for the total sample (3 months—74 years), with values ranging from 871 kcal/day (P5) to 2661 kcal/day (P95). This wide variability is consistent with the broad age range of the sample and the differences in sex and body weight among the individuals. Males had a higher energy intake than females (by approximately 313 kcal/day). The mean protein intake was 66.7 g/day (2.0 g/kg bw), contributing 16% to the energy intake (En). Protein from animal sources accounted for 62% of the total protein intake, with an animal/plant ratio of approximately 2:1.

In the total sample, and in both sexes, the total fat intake was 38% En, which exceeds the DRVs upper limit of 30% En. Intake of SFAs was also above the recommended threshold of 10% En, at 12%.

Carbohydrates provided 45% En, 19% of which derived from total sugars. The mean starch intake was 107.8 g/day. The total carbohydrate intake (in both g/day and % En) was within the recommended range (45–60%), but towards the lower limit. While carbohydrates were adequate in terms of their percentage of energy contribution, they were skewed towards a high total sugar consumption, suggesting a limited intake of complex carbohydrates. Dietary fibre intake was suboptimal at a mean of 14.6 g/day, with an energy-adjusted intake of 8.8 g/1000 kcal/day (the recommended intake is 8.4–16.7 g/1000 kcal from childhood to adulthood). Several micronutrients were below optimal levels, including vitamin D, calcium, potassium, iron (9.5 ± 3.6 mg, especially for women at 8.7 ± 3.0 mg), and folate. Adequate intake levels were found for the following nutrients: vitamin A (758.3 ± 293.9 µg RE), vitamin B12 (4.3 ± 2.3 µg), vitamin C (101.1 ± 53.4 mg), vitamin E (10.8 ± 4.4 mg), magnesium (254.1 ± 114.8 mg), and zinc (9.1 ± 3.3 mg).

**Figure 1:**
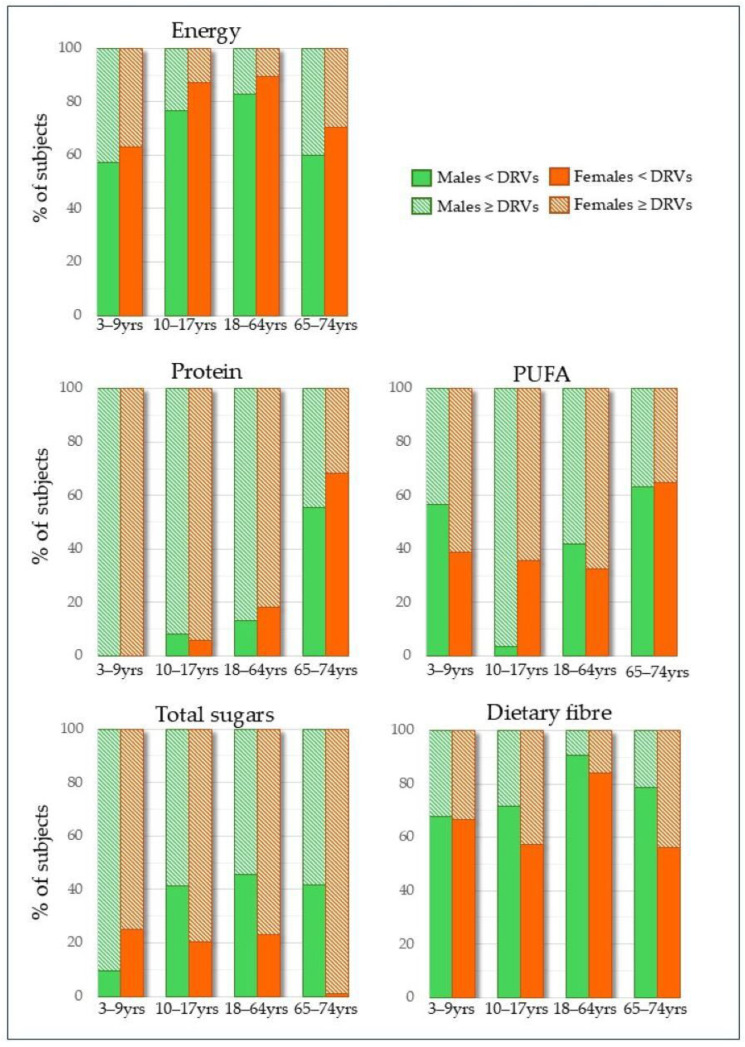
Percentage of subjects with inadequate intake of selected nutrients by age groups and sex.

Children and adolescents (Table 3 and Table 4) were in line, on average, with the reference range for energy in both sexes, and 24% and 12% of them, respectively, had intakes above the DRVs (Figure 1), as well as for protein, vitamin A, and vitamin B12. Children, in particular, consumed significantly more protein than is recommended. In absolute terms (g/day), they consume more than twice the DRVs, and when adjusted for body weight, this figure approaches three times the recommended intake per kilogram. Moreover, more than half of the children exceeded the RI (reference intake range) for the percentage of energy derived from the total fats (73% males and 72% females), as well as having intakes exceeding the SDT (suggested dietary target) for total sugar and saturated fats (Figure 1). Meanwhile, the percentage of energy derived from carbohydrates was found to be in line with the SDT (67% males and 63% females). However, it was close to the lower limit.

**Table 3:** The usual intake of energy, macronutrients, and micronutrients from food and beverages in children (3–9 years) by sex, compared with the Italian dietary reference values (DRVs).

	Males (*n* = 168)								Females (*n* = 171)							
	Mean	SD ^(d)^	P5	P25	P50	P75	P95	DRVs ^(e)^	Mean	SD ^(d)^	P5	P25	P50	P75	P95	DRVs ^(e)^
**Energy (kcal/day)**	1519.3	341.0	1066.6	1274.4	1495.4	1717.7	2144.5	1380–1790 ^(f)^	1413.9	217.0	1077.1	1242.5	1414.8	1564.1	1741.6	1280–1650 ^(f)^
**Energy (MJ/day)**	6.36	1.43	4.46	5.33	6.26	7.19	8.97	5.77–7.49 ^(f)^	5.92	0.91	4.51	5.20	5.92	6.54	7.29	5.36–6.90 ^(f)^
**Protein (g/day)**	58.6	12.3	40.9	50.6	57.5	65.7	83.1	14.8–29.3	54.5	8.1	41.5	49.3	53.8	60.5	68.2	14.6–29.7
*Protein (g/kg bw)*	2.6	0.8	1.5	2.1	2.6	3.2	4.1	0.97–0.99	2.4	0.6	1.5	2.0	2.4	2.8	3.3	0.97–0.99
*Animal protein (g/day)*	39.0	9.8	24.8	32.4	37.9	45.3	55.9		36.2	6.6	25.3	32.2	35.5	40.7	47.9	
*Animal protein (% on total)*	66.7	8.1	52.9	62.4	67.4	72.3	77.9		67.0	6.2	57.5	63.1	66.7	71.0	76.7	
**Fats (g/day)**	63.3	16.1	41.0	50.4	62.6	74.4	90.9		59.7	10.7	43.5	51.0	59.2	66.5	76.8	
*SFAs ^(a)^ (g/day)*	21.4	6.4	13.1	16.5	20.5	26.0	31.6		20.2	4.0	13.4	17.5	20.1	22.8	27.4	
*MUFAs ^(b)^ (g/day)*	28.0	6.6	18.5	23.1	27.8	32.2	38.9		26.0	4.6	18.1	23.1	26.2	28.8	33.3	
*PUFAs ^(c)^ (g/day)*	8.5	2.4	5.3	6.8	8.0	9.5	13.2		8.5	2.4	5.1	6.9	8.2	9.6	13.2	
*Cholesterol (mg/day)*	250.7	72.9	148.4	205.2	234.6	292.1	387.3		221.3	18.1	191.1	209.3	220.2	232.7	254.2	
**Carbohydrates (g/day)**	189.3	46.5	127.8	154.6	183.7	218.7	263.5		174.0	29.2	128.6	157.3	171.5	195.4	223.5	
*Starch (g/day)*	101.2	26.3	65.9	81.8	99.2	119.1	145.2		94.2	19.5	62.5	80.3	93.0	106.9	126.5	
*Total sugars (g/day)*	76.6	26.6	37.6	58.8	74.5	88.3	123.9		69.6	21.6	37.3	53.9	66.7	82.4	110.3	
*Dietary fibre (g/day)*	11.6	4.2	6.1	8.8	11.1	13.7	18.6		10.9	3.0	6.5	8.6	10.4	12.6	17.2	
*Dietary fibre (g/1000 kcal/day)*	7.7	2.2	4.6	6.2	7.5	8.9	11.0	8.4 ^(i)^	7.8	2.1	5.1	6.5	7.5	8.9	12.1	8.4 ^(i)^
**% Total energy from**																
Protein (% En)	16	2	13	15	16	17	19	10–15	16	0.0	15	16	16	16	16	10–15
Fat (% En)	37	4	32	35	37	40	44	20–35 ^(g)^	38	4	32	35	38	40	43	20–35 ^(g)^
SFAs ^(a)^ (% En)	13	2	10	11	13	14	16	<10 ^(h)^	13	2	10	12	13	14	15	<10 ^(h)^
MUFAs ^(b)^ (% En)	17	2	14	15	16	18	20		17	2	13	15	17	18	19	
PUFAs ^(c)^ (% En)	5	1	4	5	5	5	6	5–10 ^(g)^	5	1	4	5	5	6	7	5–10 ^(g)^
Carbohydrates (% En)	47	4	40	44	47	50	53	45–60 ^(g)^	46	4	40	44	46	49	52	45–60 ^(g)^
Total sugars (% En)	21	4	14	17	20	23	29	<15 ^(h)^	20	6	12	15	20	24	29	<15 ^(h)^
**Vitamins**																
*Vitamin A (REs µg/day)*	642.0	233.8	278.5	482.3	609.4	765.9	1081.6	250–400	644.4	194.7	378.6	524.1	609.0	751.1	947.8	250–400
*β-carotene (µg/day)*	2384.5	1320.0	771.0	1476.5	2116.6	3170.0	4904.1		2343.6	985.6	1022.7	1691.9	2195.8	2919.0	4113.4	
*Total folate (µg/day)*	195.5	58.5	114.8	156.9	188.6	231.2	289.0	120–200	182.7	37.8	130.8	156.8	179.6	205.2	251.1	120–200
*Dietary folate equivalent (µg/day)* ^(l)^	200.5	62.9	114.7	157.5	190.7	238.5	312.3		187.0	41.7	130.1	157.2	183.3	212.2	259.1	
*Vitamin B12 (µg/day)*	3.4	1.2	1.8	2.7	3.2	4.1	5.3	1.4–2.5 ^(i)^	3.4	1.0	2.2	2.9	3.4	3.8	4.6	1.4–2.5 ^(i)^
*Vitamin C (mg/day)*	71.8	37.0	28.0	44.9	61.5	96.2	142.6	35–60	71.3	31.7	25.9	50.3	66.3	86.6	130.7	35–60
*Vitamin D (µg/day)*	1.7	0.6	0.9	1.3	1.6	1.9	2.5	15	1.7	0.5	0.9	1.4	1.7	2.0	2.6	15
*Vitamin E (mg/day)*	8.8	2.0	5.8	7.3	8.7	9.8	13.1	5–8 ^(i)^	8.5	1.9	5.7	7.3	8.3	9.7	11.7	5–8 ^(i)^
**Minerals**																
*Calcium (mg/day)*	702.0	207.9	376.5	575.1	697.1	808.8	1018.0	510–1040	631.4	171.9	383.6	504.2	624.9	731.1	978.8	510–1040
*Iron (mg/day)*	7.7	1.9	4.7	6.5	7.6	8.6	11.4	8–13	7.1	1.3	5.3	6.1	6.9	8.1	9.3	8–13
*Magnesium (mg/day)*	199.6	47.2	131.2	168.5	193.1	227.7	285.0	120–220 ^(i)^	190.8	39.5	136.4	163.4	188.7	213.0	254.6	120–220 ^(i)^
*Potassium (mg/day)*	2040.5	581.8	1199.8	1627.7	1995.9	2365.0	3045.3	1900–2700 ^(h)^	1939.5	373.7	1354.7	1672.1	1903.7	2149.9	2640.8	1900–2700 ^(h)^
*Zinc (mg/day)*	7.8	1.6	5.3	6.6	7.7	8.7	10.4	5–8	7.2	1.1	5.5	6.5	7.2	8.0	9.0	5–8

^(a)^ SFAs: Saturated fatty acids; ^(b)^ MUFAs: mono-unsaturated fatty acids; ^(c)^ PUFAs: poly-unsaturated fatty acids; ^(d)^ SD: standard deviation; ^(e)^ DVRs: Italian dietary reference values—values reported as reference level ranges (from 3 to 9 years) expressed as PRI (population reference intake) unless otherwise indicated; ^(f)^ range of reference energy intake levels, expressed as AR (average requirement) and according to a PAL (physical activity level) of 1.6; ^(g)^ range of reference intake levels expressed as RI (reference intake range for macronutrients); ^(h)^ range of reference intake levels expressed as STD (suggested dietary target); ^(i)^ range of reference intake levels expressed as AI (adequate intake); and ^(l)^ expresses the folate content of foods by considering the different bioavailability of naturally occurring folate and added folic acid.

**Table 4:** The usual intake of energy, macronutrients, and micronutrients from food and beverages in adolescents (10–17 years) by sex, compared with the Italian dietary reference values (DRVs).

	Males (*n* = 138)								Females (*n* = 138)							
	Mean	SD ^(d)^	P5	P25	P50	P75	P95	DRVs ^(e)^	Mean	SD ^(d)^	P5	P25	P50	P75	P95	DRVs ^(e)^
**Energy (kcal/day)**	2230.1	477.4	1508.9	1934.6	2226.7	2456.4	2996.2	1900–2870 ^(f)^	1801.3	377.4	1213.6	1544.2	1773	2041.2	2415.9	1170–2310 ^(f)^
**Energy (MJ/day)**	9.33	2.00	6.31	8.09	9.32	10.28	12.54	7.95–12.01 ^(f)^	7.54	1.58	5.08	6.46	7.42	8.54	10.11	4.9–9.67 ^(f)^
**Protein (g/day)**	92.7	23.9	58	77.2	90.1	105.1	144.8	32.2–61.5	73.6	12.3	54	64.2	72	82.5	96.4	33.3–50.7
*Protein (g/kg bw)*	1.6	0.5	0.9	1.2	1.6	1.9	2.6	0.98–0.93	1.4	0.4	0.9	1.1	1.4	1.6	2.1	0.98–0.90
*Animal protein (g/day)*	62.2	21.1	32	47.2	59	72	102.7		48.6	10.5	32	41.8	47.6	56.6	67.2	
*Animal protein (% on total)*	66.2	7.6	52.2	61.6	66.5	70.5	78.0		65.8	7.3	52.8	61.2	66.1	70.8	76.3	
**Fat (g/day)**	98	25.1	60	78.9	96	112.3	140.8		76.9	16.3	53.7	65	74.8	85.3	106.4	
*SFAs ^(a)^ (g/day)*	32	8.9	18.7	25.6	31.6	37.4	48.6		25.2	5.6	16.5	21.6	25	28.8	36.1	
*MUFAs ^(b)^ (g/day)*	44.3	12.6	25.3	35.3	42.1	51.3	68		34.3	6.4	24.2	29.6	33.4	38.5	45.8	
*PUFAs ^(c)^ (g/day)*	13.8	3.6	8.7	11.8	13.4	15.7	21.2		11.1	3.9	6.1	8.6	10.6	12.5	18.3	
*Cholesterol (mg/day)*	356.9	122.1	194.7	269.7	337.9	421.9	585.8		295	34.5	234.5	270.1	295.4	314.1	351.6	
**Carbohydrates (g/day)**	257.5	58.7	166.1	213.4	256.1	291.7	370.1		213.9	54	129.5	175	209.8	252.5	308.8	
*Starch (g/day)*	155.4	38.9	94	132.5	152.1	176.8	222.9		123.3	29	75.4	104.7	125.8	142.3	170.7	
*Total sugars (g/day)*	86.8	25	51.1	67.9	82	99.8	135.9		78.9	25.1	45.2	59.7	73.5	95.1	129.1	
*Dietary fibre (g/day)*	16.4	5.1	9.1	12.8	15.6	19.3	26		14.5	4.2	8.3	11.3	14.1	17.3	22.4	
*Dietary fibre (g/1000 kcal/day)*	7.5	2.3	4.7	5.8	7.3	8.7	12.1	8.4 ^(i)^	8.3	2.2	5.1	6.8	8.1	9.6	11.8	8.4 ^(i)^
**% Total energy from**																
Protein (% En)	17	2	13	15	17	18	21	15	17	2	14	15	16	18	20	15
Fat (% En)	39	4	33	37	39	41	46	20–35 ^(g)^	38	4	32	36	38	40	45	20–35 ^(g)^
SFAs ^(a)^ (% En)	13	2	10	12	13	14	15	<10 ^(h)^	12	2	9	11	12	14	16	<10 ^(h)^
MUFAs ^(b)^ (% En)	18	3	13	16	18	19	23		17	2	14	16	17	18	20	
PUFAs ^(c)^ (% En)	6	0	5	5	5	6	6	5–10 ^(g)^	5	1	4	5	5	6	8	5–10 ^(g)^
Carbohydrates (% En)	44	6	34	41	44	47	52	45–60 ^(g)^	45	4	36	42	45	48	52	45–60 ^(g)^
Total sugars (% En)	16	4	10	13	16	18	22	<15 ^(h)^	18	3	14	16	17	19	23	<15 ^(h)^
**Vitamins**																
*Vitamin A (REs µg/day)*	762.7	282.3	377.9	577.8	704.8	941.6	1262.1	400–750	701.2	240	366.2	537	668.5	859.7	1087.4	400–650
*β-carotene (µg/day)*	2892.1	1624.6	1014.6	1641.6	2456	3750.1	5986		2762.3	1221	1110.8	1965	2689.3	3305.7	4910.3	
*Total folate (µg/day)*	280.5	85.6	169.9	216.8	261.4	327.7	432.6	270–330	238.6	64.4	149.7	196.7	232.6	271.8	364.8	270–330
*Dietary folate equivalent (µg/day)* ^(l)^	286.7	90	171.4	220.8	264.5	335.1	452.7		244.3	67.4	151.3	198.6	237.5	274.7	367.7	
*Vitamin B12 (µg/day)*	5.7	1.6	3.4	4.5	5.5	6.4	9.2	2.5–4 ^(i)^	4.6	1.3	2.9	3.9	4.5	5.2	7.1	2.5–4 ^(i)^
*Vitamin C (mg/day)*	90.2	44.2	33.3	58.1	78.9	115.3	181.8	60–105	86.9	45.3	30.5	56	77.4	113.8	163.4	60–85
*Vitamin D (µg/day)*	2.9	1.6	1.2	1.9	2.5	3.5	5.8	15	2.1	0.4	1.4	1.8	2	2.3	2.8	15
*Vitamin E (mg/day)*	13.3	3.8	7.8	10.5	12.6	15.9	20.3	8–13 ^(i)^	10.9	3.3	6.5	8.6	10.5	13	17.3	8–12 ^(i)^
**Minerals**																
*Calcium (mg/day)*	868.3	244.8	503.4	701.1	856.2	1018.5	1217.9	1040–1150	708.8	169	431.5	582	697.7	832.4	1019.3	1040–1150
*Iron (mg/day)*	11.5	2.7	7.6	9.5	11.5	13.2	16.1	13	9.8	2.1	6.4	8.5	9.9	11.1	14.1	13–18
*Magnesium (mg/day)*	294.5	65.1	202.5	250.6	288	335.9	398.7	220–380 ^(i)^	248.8	56.2	169.6	203.9	244	288.4	350	220–310 ^(i)^
*Potassium (mg/day)*	2886.3	639.6	1745.3	2499.5	2830.9	3320.9	3924.1	4500 ^(h)^	2494	562.9	1677.8	2086	2428.2	2780	3559.4	4500 ^(h)^
*Zinc (mg/day)*	12.0	3.3	7.4	9.4	11.7	13.7	18.8	8–12	9.8	2.2	6.5	8.2	9.7	11.1	13.8	8–9

^(a)^ SFAs: Saturated fatty acids; ^(b)^ MUFAs: mono-unsaturated fatty acids; ^(c)^ PUFAs: poly-unsaturated fatty acids; ^(d)^ SD: standard deviation; ^(e)^ DVRs: Italian dietary reference values—values reported as reference level ranges (from 10 to 17 years), expressed as PRI (population reference intake) unless otherwise indicated; ^(f)^ range of reference energy intake levels, expressed as AR (average requirement) and according to a PAL (physical activity level) of 1.6; ^(g)^ range of reference intake levels expressed as RI (reference intake range for macronutrients); ^(h)^ reference intake levels, expressed as STD (suggested dietary target); ^(i)^ range of reference intake levels, expressed as AI (adequate intake); and ^(l)^ expresses the folate content of foods by considering the different bioavailability of naturally occurring folate and added folic acid.

**Figure 2:**
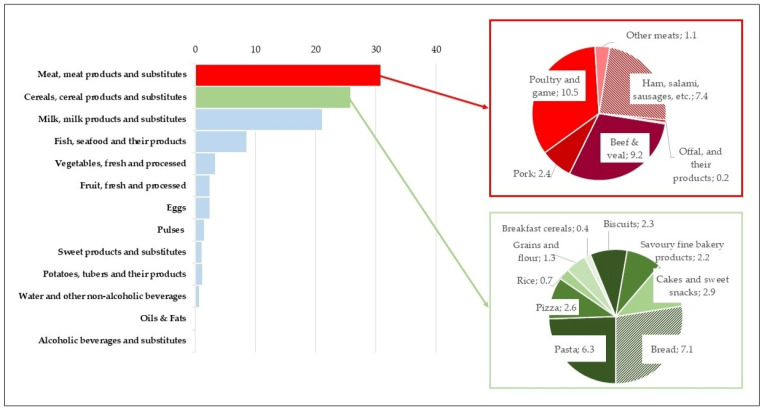
Percentage contribution of the main food categories and their subgroups to the total protein intake in total sample (*n* = 1969).

A significant proportion of children and adolescents had intakes below the DRVs for other nutrients. For instance, the prevalence of inadequacy was high for vitamin D, with extremely low intake levels observed across all sex and age groups. Additionally, almost all females aged 10–17 years had an insufficient iron and calcium intake (94% and 100%, respectively). The intake of vitamins C and E, total folate (excluding adolescent females), magnesium, calcium, and zinc was generally adequate for all children and adolescents. However, dietary fibre intake levels were below the established adequate intake (AI) (about 67%, regardless of gender or age group), only coming close to the recommended level for female adolescents (8.3 g/1000 kcal vs. 8.4 g/1000 kcal DRV).

Table 5 and Table 6 show the nutrient intake of adult and elderly males and females, respectively. A high proportion of adults of both sexes had intakes below the DRVs for energy, vitamin D, and calcium. Notably, 76% of male adults and 85% of females did not meet the DRVs for energy and 100% for vitamin D (both sexes), while over 75% did not meet the DRV for calcium (up to 84% in females). Furthermore, 95% of females aged 18–64 years had an inadequate iron intake, with observed P50 and P95 values of 10.3 and 14.2 mg/day, respectively, compared to the recommended daily value of 18 mg/day for women of childbearing age. The adequate intake for dietary fibre was only achieved by 12% of adults (Figure 1), regardless of sex.

Inadequate nutrient intake was more prevalent among younger age groups than older ones. For example, compared to elderly individuals, a greater proportion of adults had percentages of energy intake from protein, fat, and total sugars that exceeded the DRVs, RI, or adequate intake (AI) (Figure 1). A total of 55% of elderly females reached the recommended minimum target for the percentage of energy derived from carbohydrates. Among adults of both sexes and elderly males, however, the figure was much lower, at 27%.

In addition, the elderly population showed adequate intakes of protein (g/kg bw) and polyunsaturated fatty acids, as well as of antioxidant vitamins (A, C, E), vitamin B12, folate, iron, zinc, and dietary fibre (in females only). Ultimately, they had a severe vitamin D deficiency (100% in both sexes), which is common among the elderly, as well as an inadequate calcium intake (88% males and 97% females). The same applied to adults.

The percentage of individuals whose intake exceeded the UL values for calcium, zinc, vitamins A, E, and D, and folic acid has also been calculated. Only an adult and an elderly woman had values above the UL threshold for vitamin A, and only four adolescent males exceeded the UL value for zinc. A total of 12% of children (aged 3–10 years) had a zinc intake that was higher than the UL value and 1.5% had a vitamin A intake higher than the UL value.

### 3.2. Major Dietary Sources of Nutrients

Table 7 and Table 8 show the percentage contribution of the 15 main food groups to the total energy, macronutrients, and micronutrients for the total population.

**Figure 3:**
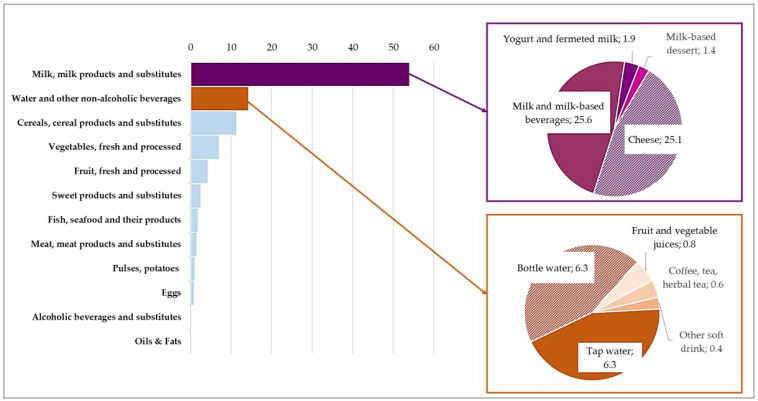
Percentage contribution of the main food categories and their subgroups to total intake of calcium in total sample (*n* = 1969).

**Figure 4:**
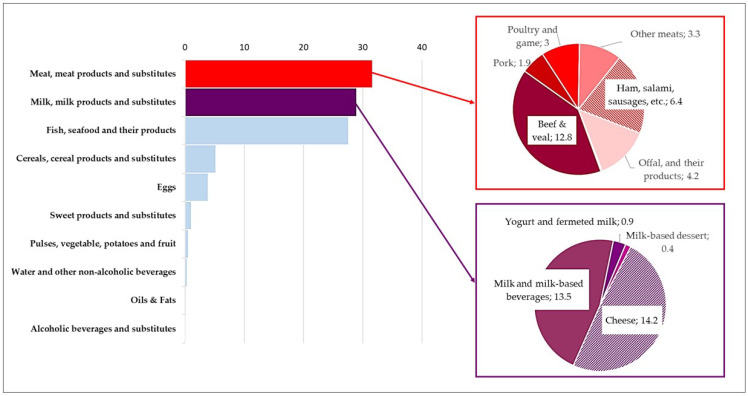
Percentage contribution of the main food categories and their subgroups to total intake of vitamin B12 in total sample (*n* = 1969).

Figure 2, Figure 3 and Figure 4 focus on the percentage contribution of the main food groups and a breakdown at the subgroup level for the main categories of protein, vitamin B12, and calcium intake. The Supplementary Material (Tables S8–S14—Dietary sources) provides detailed information on nutrient sources, broken down by food group and subgroup by age classes.

#### 3.2.1. Energy, Macronutrients, and Dietary Fibre

*Cereals, cereal products, and substitutes* were the main source of energy, accounting for 38.8% of the total energy intake. The next largest contributors were *milk, milk products, and substitutes* (14.9%), *oils and fats* (12.9%) and *meat, meat products, and substitutes* (10.0%) (Table 7).

Within the cereal group, the largest energy contributors were *bread* (8.4%), *pasta* (7.8%), *cakes and sweet snacks with and without creams* (6.3%), baked goods such as *biscuits* (5.5%), and *pizza* (3.6%) (Supplementary Material Table S14—Dietary sources total population). *Fruit, fresh and processed* provided 5.8% of energy, while *vegetables, fresh and processed* and *potatoes, tubers, and their products* contributed 4.1%.

Analysis of dietary protein intake in the studied population revealed that *meat, meat products, and substitutes* were the main source of protein, providing 30.9% of the total intake.

*Cereal*-based foods followed closely behind, accounting for 25.9% of the total intake, which highlights their significance as a dietary staple (Figure 2). Together, these two food groups represented over half (56.8%) of the total dietary protein intake, reflecting their central role in the Italian dietary pattern. *Meat, meat products, and substitutes* are primarily a source of animal protein (47%). Within this category, *poultry and game* were the main contributors, providing 10.5% of the total protein intake and 15.9% of the animal protein intake (Supplementary Material Table S14—Dietary sources total population). *Beef and veal* were also major contributors, providing 9.2% of the total protein and 14.1% of the animal protein. Meanwhile, processed meats—including ham, salami, and sausages—accounted for 7.4% of the total protein intake and 11.2% of the animal protein (Supplementary Material Table S14—Dietary sources total population). In contrast, *cereals, cereal products, and substitutes* were the main source of plant proteins, accounting for 69.8% of the total plant protein intake (Table 7). Within the cereals category, *bread* alone accounted for 7.1% of the total protein and 20.4% of the plant protein; *pasta and pasta substitutes* followed, providing 6.3% and 17.1% of the total and plant proteins, respectively; sweet bakery products, such as *cakes* and *biscuits*, collectively contributed 5.2% of the total protein (Figure 2).

Oils and fats were the predominant source of the total fat intake, accounting for 33.7%. A further contribution of 21.1% came from the *milk, milk products, and substitutes* and *meat, meat products, and substitutes categories*, with 21.1% and 13%, respectively (Table 7).

Cereals were the main source of carbohydrates, accounting for 62.5% of the total intake and for 22.7% of the total sugars. *Bread* and *pasta* contributed 15.1% and 14.2% to the carbohydrates, respectively, and 2.2% and 1.7% to total sugars (Supplementary Material Table S14—Dietary sources total population). *Fruit* contributed 9.3% to carbohydrates and a substantial 21.8% of the total sugars. *Sweet products and substitutes* followed, with 7.2% and 16.6% of the carbohydrates and total sugars, respectively. Sweet bakery products, such as *biscuits* and *cakes*, contributed significantly to the sugar intake (6.3% and 9.8%, respectively) (Supplementary Material Table S14—Dietary sources total population).

Cereals were the most significant source of dietary fibre, accounting for 42.3% of the total. Fruit and vegetables were also key contributors, accounting for 23.8% and 19.1%, respectively.

#### 3.2.2. Minerals and Vitamins

The majority of the calcium intake came from *milk, milk products, and substitutes*, accounting for 54% of the total (Figure 3). Within this category, *cheese* and *milk* were the main contributors, providing 25.1% and 25.6% of the dietary calcium, respectively. It should be noted that 14.3% of the calcium intake came from *water and other non-alcoholic beverages*, particularly tap water and bottled water, which contributed 12.6%.

Iron intake is largely provided by *cereals* (34.2%), particularly *bread* and *pasta* (9.2% and 7.5, respectively), followed by *meat* (14.9%) and *vegetables* (11.9%) (Table 8 and Supplementary Material Table S14—Dietary sources total population).

*Cereals* were the main dietary source of magnesium, accounting for 25.7% of the total intake, and they also supplied a significant proportion of potassium (14.1%). This dual contribution highlights the nutritional importance of cereals in ensuring an adequate intake of both minerals. Similarly, *vegetables* and *fruits* contributed to both micronutrients, providing 9.7% and 9.8% of the magnesium, respectively, and 16.1% of the potassium. These two food groups were key suppliers of potassium and made a meaningful contribution to the magnesium intake.

Zinc intake appears to be evenly distributed among *meat* (24.3%), *milk, milk products, and substitutes* (23.3%), and *cereals, cereal products, and substitutes* (22.0%) (Table 8). However, given that zinc from plant sources may be less bioavailable due to its phytate content, the significant contribution of animal-based sources such as *cheese* (13.8%) and *beef* (11.4%) is nutritionally relevant (Supplementary Material Table S14—Dietary sources total population).

*Vegetables, fresh and processed* were a significant source of multiple micronutrients in the diet. They were the leading contributors to vitamin A intake (43.4%), largely due to their high β-carotene content, which is a provitamin A compound. In fact, *vegetables* accounted for an overwhelming 69.7% of the total β-carotene intake and provided substantial amounts of folate (24.6%). They were also the main source of vitamin C (37.6%). Other significant dietary sources of vitamin C were *fruit* (31.7%) and *fruit and vegetable juices* (13.0%) (Table 8).

*Milk, milk products, and substitutes* and *fruit* were secondary sources of vitamin A (20.0% and 11.9%, respectively), and for β-carotene, *Fruit* contributed an additional 19.1%. Furthermore, the majority of the folate intake came from *cereals* (28.5%), *milk* (12.7%), and *fruit* (9.7%) (Table 8). The prominence of cereals was likely due to a combination of natural and fortified sources.

As shown in Figure 4, the majority of the vitamin B12 intake came from animal-sourced foods, with *meat, meat products, and substitutes, milk, milk products, and substitutes,* and *fish, seafood, and their products* being the main contributors. Specifically, *meat* accounted for 31.7% of the total vitamin B12 intake, and the primary contributors within this category were *beef and veal* (12.8%). *Milk, milk products, and substitutes* also played a significant role, accounting for 29.0% of the total B12 intake. This was largely due to *cheese* (14.2%) and milk, milk-*based beverages and substitutes* (13.5%). *Fish* accounted for an additional 27.6% of the total vitamin B12 intake.

The highest sources of vitamin D intake were *fish, seafood, and their products* (32.3%), followed by *milk* (25.4%), and *meat* (17.1%). Meanwhile, vitamin E intake was primarily associated with *oils and fats* (46.2%), followed by *vegetables* (11.4%), *fruit* (10.1%), and *milk, milk products, and substitutes* (7.1%) (Table 8).

## 4. Discussion

The IV SCAI survey, involving 1969 Italian participants aged three months to 74 years, provides a current and comprehensive overview of dietary intake across the Italian population. The results for dietary intake reveal a nutritional imbalance, as the qualitative composition of the diet differs from both established DRVs and the traditional Mediterranean dietary model.

### 4.1. Energy and Nutrient Assessment

Overall, these data suggest a dietary profile that is characterised by relatively low-calorie content, particularly among adults, with the mean energy intake for both sexes falling below the dietary reference values. In children and adolescents, the mean energy intake appears to be adequate for both sexes. However, while the value is adequate for elderly males, it is insufficient for elderly females, particularly those in the lower intake percentiles. This suggests an underestimation or reduction in energy intake, mainly among older females. This was confirmed by estimating the basal metabolic rate (BMR) by using the Harris–Benedict predictive equation [24], which takes into account weight, height, age, and sex. This value was then compared with the average energy intake over the two-day study period to obtain the PAL (physical activity level) value. The results indicate a low PAL value for adolescents, adults, and elderly women (PAL 1.3). For children aged 3–9 years and older men, the values are slightly higher (PAL range 1.4–1.5).

Across all age groups, protein intake consistently and substantially exceeds the dietary reference values. This excess is almost three times the recommended value in childhood and 40–60% in adulthood. This excess is evident in both the total amount of protein consumed and the proportion derived from animal sources (66% of total protein intake), suggesting a lack of diversity in protein sources. Such sustained overconsumption of protein, particularly from animal sources, could be associated with increased adiposity and metabolic risk later in life [54,55]. The quality of fat also represents a major concern: total fat and saturated fatty acids consistently exceed the recommended limits in every age group, accounting for 36–39% and 10–13%, respectively, of total daily energy intake, depending on the group. Meanwhile, the intake of polyunsaturated fatty acids remains at the lower end of the desirable range, indicating an imbalance in fat quality. Although the carbohydrate intake sometimes falls within the recommended range during childhood, there is a clear downward trend throughout adolescence and into adulthood and old age.

At the same time, the total sugar consumption often exceeds the desirable thresholds (typically >15% of energy intake, with up to 20% in children of both sexes), reflecting a high consumption of sugary drinks, snacks, sweets, and similar foods. Dietary fibre intake is uniformly low. All age groups show fibre densities below the recommended levels, implying inadequate consumption of fruits, vegetables, whole grains, pulses, and other plant-based foods [28].

A comparative analysis of micronutrient intake across different age groups reveals common deficiencies and age-specific patterns. A consistent finding throughout all life stages is vitamin D deficiency, with average intakes ranging from 1.7 µg/day in children to 2–3 µg/day in adolescents and adults and 2.0–2.6 µg/day in older adults, compared with the DRV of 15 µg/day. Similarly, calcium intake remains below the recommended levels in almost all groups, particularly among females (children, adolescents, and women of reproductive and postmenopausal age). This widespread insufficiency has been repeatedly documented in the literature [56] and highlights a major public health concern with potential implications for bone metabolism, immune function, and overall health [57]. There is a clear trend in iron intake, depending on sex and age. Deficiency is most prevalent among females during adolescence and their reproductive years, which could impact their haematological status and fertility [58].

Conversely, adequate iron levels are observed in older adults, likely due to reduced requirements following the menopause. Among adolescents, notably low potassium intake (approximately 2494–2886 mg per day versus a DRV of 4500 mg per day) suggests limited consumption of fruit and vegetables. However, magnesium and zinc intake is generally adequate in both sexes. The overall vitamin profile in adults and the elderly is generally adequate, including vitamins A, C, E, and B12. However, calcium, magnesium, and potassium intake remains suboptimal, highlighting ongoing nutritional vulnerabilities in these groups.

Comparing the previous INRAN-SCAI national survey (2005–06) with the IV SCAI survey (2017–20) reveals substantial changes in the dietary patterns of the Italian population over approximately fifteen years [13,40,59]. Overall, the data suggest a general decline in total energy and nutrient intake, accompanied by relatively stable macronutrient distribution, but a modest decrease in diet quality and micronutrient density. Considering the total population of both surveys, the mean daily energy intake decreased markedly from approximately 2100 kcal/day in 2005–06 to 1667 kcal/day in 2017–20: a decrease of 21%. This reduction aligns with broader European trends indicating a decline in caloric intake, which is likely due to lower energy expenditure and sedentary lifestyles [60,61,62]. However, the differences in estimated intakes may also reflect methodological inconsistencies between the two national surveys, which could lead to underreporting. The INRAN-SCAI survey was based on household-level data and three consecutive food diaries for each member of the household, whereas the IV SCAI survey adopted an individual-level approach involving two non-consecutive 24 h recalls or food diaries, organised by age group [28], which introduced greater variability in mean intake estimates. Despite the decrease in calories, the distribution of macronutrient energy remained stable: protein accounted for around 16% of the total energy in both surveys, fat increased slightly from 36% to 38%, and carbohydrates remained at around 45%. However, when expressed in absolute terms, the mean intakes of proteins (from 82 g/day to 66.7 g/day), fats (from 84.5 g/day to 70.5 g/day), and carbohydrates (from 256.4 g/day to 194.3 g/day) each declined by approximately 15–20%. The fat quality profile also showed limited improvement: saturated fatty acids increased slightly (from 11.3% to 12% of energy), remaining above the recommended threshold (<10% En), while monounsaturated and polyunsaturated fatty acids remained stable. Furthermore, dietary fibre decreased from 18.2 g/day in 2005–06 to 14.6 g/day 2017–20, despite carbohydrate energy contributions remaining similar.

Overall, the intakes of most minerals showed a downward trend between the two surveys. Potassium, magnesium, zinc, and iron decreased by approximately 10–20%, while calcium intake (from 760 to 733.6 mg/day in 2005–06 and 2017–20, respectively) remained consistently below the recommended levels. These reductions likely reflect the decline in plant-based food consumption, particularly fruit, vegetables, and legumes [28]. Similarly, vitamin intakes exhibited a general decline: vitamin C dropped by about 20%, and vitamins E and B12 decreased by 12% and 27%, respectively. In contrast, the vitamin A and β-carotene intakes remained relatively stable, whereas the vitamin D intake persisted at critically low levels (2.3–2.6 µg/day), well below the Italian adequate intake (15 µg/day).

Similar inadequate intake of several vitamins, minerals, and macronutrients has also been reported in many countries. National dietary surveys in Finland [63], Ireland [64], Serbia [65], Slovenia [66], and Spain [67] found that protein and total fat intake generally met or exceeded recommendations, while dietary fibre and key micronutrient intake remained below optimal levels. Excessive consumption of saturated fatty acids and insufficient intake of vitamins D and E, folate, and calcium were consistent findings across countries. In Finland, the FinDiet 2017 Survey (*n* = 1655; aged 18–74) found that protein intake was higher than necessary, with close to 70% derived from animal sources. Total fat and polyunsaturated fatty acid intakes were adequate, but saturated fatty acid and salt intakes exceeded recommendations across all socioeconomic groups [63]. Similarly, the Serbian National Survey of Adults (18–64 years) revealed that, while the mean protein intake was within the recommended range, around 17% of adults (mostly women) did not reach the minimum recommended by the European Food Safety Authority (EFSA) of 0.83 g/kg/day. Furthermore, over 90% of the population exceeded the upper limits for total fat intake [65]. The Slovenian SI.Menu 2017/18 study also revealed that energy from proteins was mostly sufficient, whereas carbohydrate intake was below the recommendations, particularly among adults and males. About one quarter of participants exceeded the recommended total fat intake, while fibre intake was inadequate in more than 85% of adults and elderly people [66]. These trends are consistent with those observed in Finland and Serbia, confirming a continental pattern of high-fat and low-fibre consumption. The Irish Children’s Food Survey II (2017–2018) revealed moderate improvements compared to the 2003–2004 survey, including reductions in SFA (from 15% En to 14%En) and free sugars (from 16%En to 9% En), as well as an increase in fibre intake (from 12 to 15 g/day). Nonetheless, mean intakes of SFA and free sugars still exceeded WHO recommendations (<10% En and <5% En, respectively) [64].

A similar macronutrient imbalance was observed in children and adolescents in Spain in the ENALIA 2013–2014 Survey, including excessive protein intake and a high energy contribution from SFAs (>10% En), at the expense of PUFAs. Although the fat quality improved compared to earlier data (EnKid 1998–2000), intake of SFAs remains a major concern for cardiovascular health [67].

All studies reported micronutrient inadequacies. In Finland, low intakes of vitamins A, D, C, B1, B2, and folate, as well as calcium, were observed across socioeconomic strata [63]. Despite overall energy adequacy, Irish children showed inadequate intakes of vitamin D, calcium, iron, and folate [64]. In Serbia and Slovenia [65,66], it was the elderly population who were particularly vulnerable. In Slovenia, for example, more than half of the elderly participants (70.9% of males and 56.7% of females) failed to achieve the recommended daily protein intake of 1.0 g/kg, raising concerns about sarcopenia and frailty [66].

### 4.2. Food Source Assessment

The Italian dietary pattern is still based mainly on cereals and cereal-based products, accounting for almost 40% of the total energy intake. They are also the main source of carbohydrates (62.5%) and dietary fibre intake (42.3%), with bread and pasta being the primary sources. This finding is consistent with the traditional Mediterranean dietary model, which emphasises grains, particularly bread and pasta, as the main sources of energy [68,69]. Milk and dairy products, together with oils and fats, are the second and third largest contributors to total energy intake.

More than half of the total protein consumption derives from meat (30.9%) and cereal-based foods (25.9%), which together provide the largest proportion of protein intake. Meat, particularly poultry, beef, and processed meats, are the main source of animal protein, while cereals account for almost 70% of the total plant protein. This balance highlights the coexistence of traditional and modern dietary behaviours: animal proteins ensure high biological value and bioavailability, while plant-based sources support dietary diversity and sustainability. On the other hand, the observed diet continues to exhibit a relatively high ratio of animal-to-plant protein intake (67% versus 33%). The current literature suggests that dietary patterns with approximately 50% of their total protein from plant sources and 50% from animal sources succeed in combining lower environmental impact with adequate nutritional coverage [70,71,72]. A recent study [73] of a European population (40,101 adults, aged 18–64 years) showed that the dietary pattern defined as “plant-forward” provided approximately 52% of the total protein intake from animal sources (and therefore ~48% from non-animal sources) and was associated with the highest nutritional quality and the lowest environmental impact (in terms of greenhouse gas emissions and land use) compared with the average. In contrast, patterns with more animal proteins (≈64–69% animal protein) were common in most countries but were associated with a higher environmental impact.

Oils and fats are the main source of the total lipids, significantly contributing to MUFAs and PUFAs intake. Nevertheless, dairy and meat products are a substantial source of SFA and cholesterol, with cheese, eggs, and processed meats being the main contributors.

Vegetables were the dominant contributors to vitamin A (43.4%) and β-carotene (69.7%) intake, as well as being a key source of vitamin C (37.6%) and folate (24.6%).

In terms of micronutrients, calcium intake is primarily derived from milk and dairy products (54%), with water and other non-alcoholic beverages also representing a significant source (14.3%). This highlights the importance of water in achieving an adequate calcium intake. Iron intake largely comes from cereals (34.2%), followed by meat (14.9%) and vegetables (11.9%). However, the prevalence of plant-based iron (non-haem iron) raises concerns about bioavailability, particularly among populations with increased iron requirements, such as menstruating women. Although red meat contributes valuable haem iron, its proportion of the total intake is small. Animal-based foods such as meat, dairy products, and fish are almost the only sources of vitamins B12 and D, while zinc is evenly distributed among meat, dairy products, and cereal products.

A comparative analysis of two national surveys reveals a high degree of consistency in the overall dietary patterns observed in the Italian population. Both the INRAN-SCAI survey [59] and the IV SCAI survey confirm that cereals, cereal products, and their substitutes remain the staple of the Italian diet. These foods represent approximately 38–39% of the total energy intake, respectively, and provide the largest proportion of carbohydrates (66.7% in the INRAN-SCAI survey versus 62.5% in the IV SCAI survey) and dietary fibre. Within this group, bread and pasta are the main contributors to energy, protein, and carbohydrate intake. Similarly, both national studies identified milk and dairy products, meat and meat products, and oils and fats as the main sources of energy after cereals. Milk and dairy products provide around one-fifth of the total protein and fat intake (20.8% and 21.2%, respectively) and are the main source of calcium and saturated fatty acids (35.8% and 35.4%, respectively). Oils and fats account for most of the intake of monounsaturated fatty acids, while meat and meat products represent approximately one-third of the protein intake (27.2% and 30.9%) and a significant proportion of cholesterol and vitamin B12 intake (31.7% in both surveys). These patterns are consistent across both Italian national studies, confirming a dietary model that is energy-sufficient, yet heavily reliant on animal sources for protein and fat. The mineral and vitamin profile of the two surveys is consistent and stable over time: cereals provide the mineral base, while calcium and zinc come from animal foods. The central role of vegetables and fruit in providing antioxidant vitamins and of animal foods in providing fat-soluble and B vitamins is confirmed [59].

### 4.3. Implications of the Study: Strengths and Limitations

The IV SCAI (2017–2020) survey is a comprehensive and methodologically rigorous assessment of dietary intake in the Italian population. It was conducted in accordance with the European Food Safety Authority’s EU Menu guidelines [30]. The strengths of the survey included the collection of data, balanced for season and weekday; gender-specific analyses; and the use of validated tools (e.g., the food atlas) to improve the accuracy of portion-size estimation. Rigorous interviewer training and robust quality assurance procedures ensured the reliability of the data. The survey’s stratified sampling design ensured national representativeness across sex, age, and geographic regions, thereby enhancing its external validity. Standardised data collection protocols and harmonised food classification systems (FoodEx2) facilitate comparability with other European dietary surveys [44,45]. Using FoodSoft 1.0 and the associated CREA-developed food composition databases represents a major methodological advancement. This continuously updated database integrates over 3000 foods and 1500 recipes, and the nutrient database is thoroughly and comprehensively completed for each food item (no missing data). Using two non-consecutive 24 h recalls or food diaries, supported by the multiple pass method, improved data accuracy and likely minimised recall bias [74]. Furthermore, applying the multiple source method (MSM) to estimate the usual intakes accounted for intra-individual variability, yielding more reliable estimates of nutrient adequacy [48]. Finally, as previously mentioned, these findings were essential for developing the Italian Dietary Guidelines for Healthy Eating [14,75] and were also crucial for formulating the latest Reference Intake Levels of Nutrients and Energy for the Italian population [24]. These reference levels have already been updated to consider food consumption and intake assessments from the IV SCAI national survey.

Nevertheless, several methodological limitations should be acknowledged. Although the overall sample size (*n* = 1969) is representative, this may impact the accuracy of subgroup analyses. Furthermore, the exclusion of adults over 74 years limits the generalisability of the study to the elderly population. The analysis only considered nutrient intake from food, excluding dietary supplements. This may result in an underestimation of total nutrient adequacy, particularly with regard to vitamin D and calcium, for which inadequacy remains prevalent in Europe [76,77]. As with most self-reported dietary data, under-reporting of energy and socially undesirable foods is probable, particularly among women and older adults [78,79]. This is a limitation of population-based dietary assessments, as self-reported data often underestimates energy intake by 20–25% compared to actual expenditure [80,81,82]. This may be due to factors such as memory loss, incomplete reporting, difficulty estimating portion sizes, and social desirability bias, all of which can lead to the underreporting of energy-dense foods. Such misreporting affects the accuracy of population-level data on energy balance and nutritional intake [83,84].

In any case, this relevant limitation concerns the assessment of energy adequacy at an individual level and is primarily due to the lack of direct or objective measurements of physical activity or total energy expenditure. Our methodology involved comparing the energy and nutrient intake with the population-based reference values: a common and validated approach in large-scale nutritional surveys. However, it may not fully capture the inter-individual variability in energy requirements. The absence of individual physical activity data complicates the interpretation of associations between dietary intake and outcomes. Higher intakes in certain individuals may simply reflect higher energy expenditure in more active participants, or vice versa. This makes it difficult to ascertain the direction of the observed effects a priori. To explore this issue, the BMR was estimated using the Harris–Benedict predictive equations, and a PAL was indirectly derived by comparing the estimated energy requirements with the self-reported energy intake. The low PAL values observed across different age and sex groups are consistent with data from other dietary surveys. They suggest that energy intake is likely to be underestimated, particularly in populations that have a history of under-reporting, such as women and adolescents. As PAL was inferred indirectly from self-reported intake, rather than being measured independently, these estimates should be interpreted with caution and cannot be used as a substitute for objective assessments of energy expenditure. These considerations emphasise that the evaluation of adequacy in the present study should primarily be interpreted at a population level, rather than an individual level. This may serve as a warning for future studies.

## 5. Conclusions

Overall, the Italian population adheres only moderately to the Mediterranean diet. The average person’s usual intake is characterised by an excessive intake of animal proteins, saturated fats, and sugars, alongside inadequate consumption of dietary fibre, essential micronutrients, and plant-based foods. Women, in particular, have a more critical nutritional profile, with a higher prevalence of deficiencies in iron, folate, calcium, and vitamin D. Although traditional staple foods, such as cereals, fruits, vegetables, and olive oil, are still the major source of key nutrients, there has been a clear shift towards a higher intake of animal protein, saturated fat, and refined carbohydrates. While these trends reflect sociocultural changes and possible methodological biases in dietary assessment, they consistently suggest a gradual erosion of the traditional Mediterranean dietary pattern. From a sustainable perspective, the Italian diet remains outside of the 50:50 plant-to-animal protein target, highlighting the need for dietary shifts toward increased plant protein consumption to improve both environmental sustainability and nutritional balance. Hence, to preserve the health and sustainability benefits of this diet, targeted nutritional policies and educational initiatives are needed to promote the consumption of plant-based foods, pulses, whole grains, and fish, and to reduce reliance on processed and animal-derived products. This would improve overall dietary quality, address specific micronutrient deficiencies, and support healthy ageing in the population.

## Figures and Tables

**Table 1:** IV SCAI sample characteristics: height, weight and body mass index (BMI) * and BMI class * by age groups and sex.

			Weight (kg)	Height (cm)	BMI (kg/m^2^)	BMI Class (%)			
Sex	Age Groups (ys)	*n*	Mean (SD)	Mean (SD)	Mean (SD)	Underweight	Normal	Overweight	Obese
Male	3–11 (months)	75	9.2 (1.6)	71.9 (4.9)	17.8 (2.2)	1.3	66.7	21.3	10.7
	1–2	162	13.2 (2.0)	89.1 (6.9)	16.6 (1.7)	3.5	63.8	23.4	9.4
	3–9	168	23.9 (9.0)	117.0 (15.1)	17.0 (3.1)	1.2	58.5	22.1	18.2
	10–17	138	58.4 (14.3)	165.2 (13.7)	21.2 (3.7)	0.9	86.8	9.8	2.5
	18–64	346	80.7 (14.2)	176.4 (6.9)	25.9 (4.4)	0.2	49.0	37.8	12.9
	65–74	65	81.6 (13.9)	169.3 (6.6)	28.4 (4.1)	0.0	19.2	52.8	28.0
Female	3–11 (months)	75	8.3 (1.4)	70.1 (4.7)	16.7 (1.5)	1.3	84.0	10.7	4.0
	1–2	160	12.5 (2.1)	87.7 (7.1)	16.2 (2.0)	0.4	75.4	17.3	6.9
	3–9	171	23.9 (8.1)	117.7 (13.8)	16.8 (2.9)	2.0	63.7	21.2	13.2
	10–17	138	54.8 (13.1)	159.5 (8.5)	21.4 (4.2)	0.0	90.5	6.8	2.7
	18–64	380	64.2 (13)	162.9 (6.2)	24.2 (4.8)	5.1	63.1	19.7	12.2
	65–74	91	69.5 (15.6)	157.6 (7.5)	28.1 (6.7)	0.8	42.0	28.0	29.2
Total	3–11 (months)	150	8.7 (1.5)	71.0 (4.9)	17.2 (1.9)	1.3	75.3	16.0	7.3
	1–2	322	12.8 (2.1)	88.4 (7.0)	16.4 (1.8)	2.0	69.5	20.4	8.1
	3–9	339	23.9 (8.5)	117.4 (14.4)	16.9 (3.0)	1.6	61.1	21.7	15.6
	10–17	276	56.6 (13.8)	162.4 (11.8)	21.3 (3.9)	0.5	88.7	8.3	2.6
	18–64	726	72.0 (15.9)	169.2 (9.4)	25.0 (4.7)	2.8	56.5	28.2	12.5
	65–74	156	74.6 (16.0)	162.5 (9.1)	28.2 (5.8)	0.5	32.5	38.4	28.7

* Infants, school-aged children, and adolescents were classified according to BMI age- and sex-specific z-score cut-off points proposed by the WHO from birth to 5 years and 5–19 years [49,50,51], using WHO AnthroPlus software [52]. For adults and elderly people, the BMI cut-off points applied are those suggested by the WHO [53] (underweight: BMI < 18.5; normal: 18.5 ≤ BMI < 25; overweight: 25 ≤ BMI < 30; and obese: BMI ≥ 30).

**Table 2:** The usual intake of energy, macronutrients, and micronutrients from food and beverages for total population (3 months–74 years) by sex.

	Males (*n* = 954)							Females (*n* = 1015)							Total (*n* = 1969)						
	Mean	SD ^(d)^	P5	P25	P50	P75	P95	Mean	SD ^(d)^	P5	P25	P50	P75	P95	Mean	SD ^(d)^	P5	P25	P50	P75	P95
**Energy (kcal/day)**	1828.2	648.8	896.5	1311	1792	2273.5	2955.3	1515.1	419.4	863.8	1209.5	1499.8	1782.5	2251	1666.8	562.3	871.5	1237.9	1598	2017.2	2661.2
**Energy (MJ/day)**	7.65	2.71	3.75	5.49	7.50	9.51	12.36	6.34	1.75	3.61	5.06	6.28	7.46	9.42	6.97	2.35	3.65	5.18	6.69	8.44	11.13
**Protein (g/day)**	73.6	30.1	29.5	51.3	71.4	91.8	123.2	60.3	19	30.3	46.9	59.5	73.1	92.9	66.7	25.8	29.3	48.4	64.6	81.8	111.4
*Protein (g/kg bw)*	2.0	1.1	0.8	1.2	1.7	2.7	4.3	1.9	1.0	0.7	1.1	1.5	2.6	3.9	2.0	1.1	0.7	1.1	1.6	2.6	4.1
*Animal protein (g/day)*	48.8	21.2	20.3	33.8	45.8	60.3	89.0	39.4	13.4	19.2	29.6	38.6	47.8	63.5	44.0	17.9	19.3	31.4	41.5	53.8	76.1
*Animal protein (% on total)*	66.7	7.9	53.8	62.5	67.2	71.7	77.9	65.7	8.6	51.1	61.2	66.3	71.1	77.3	66.2	8.3	52.3	61.7	66.8	71.4	77.6
**Fat (g/day)**	76.8	29.2	37.3	52.9	73.8	96.1	132.3	64.6	19.2	36.0	50.0	63.4	77.7	98.4	70.5	24.9	36.3	51.6	67.5	85.2	116.8
*SFAs ^(a)^ (g/day)*	24.8	9.0	13.2	17.8	23.3	29.8	41.7	20.9	6.0	11.9	16.3	20.5	25.0	30.9	22.8	7.8	12.3	17.1	21.8	27.3	36.9
*MUFA s ^(b)^ (g/day)*	35.2	14.1	16.1	23.9	33.6	44.0	60.7	29.6	9.3	16.1	22.8	28.9	35.7	47.3	32.4	12	16.00	23.2	30.7	39.4	54.5
*PUFAs ^(c)^ (g/day)*	10.6	5.1	4.5	6.9	9.7	13.3	19.6	9.0	3.6	4.4	6.4	8.5	10.9	15.0	9.8	4.4	4.4	6.6	9.0	12.1	17.4
*Cholesterol (mg/day)*	273.8	121.3	107.4	190.9	256.3	337.8	506.9	220.6	72.7	108.1	172.5	214.0	263.5	350.4	246.4	100.7	105.8	177.6	232.8	301.1	426.6
**Carbohydrates (g/day)**	211.6	71.5	109.3	157.8	204	256	339.1	178.1	50.2	104.1	142	173.6	208.1	268.7	194.3	63.4	105.5	148.5	186.4	233.3	306.5
*Starch (g/day)*	119.7	50.3	47.5	81.8	116.1	151.1	207.7	96.7	34.6	43.7	73.3	95.5	120.9	156.4	107.8	44.2	43.6	76.6	103.5	136.1	185.6
*Total sugars (g/day)*	79.4	26.4	42.6	60.9	75.5	93.5	126.8	71.2	20.8	41.1	56.7	67.9	83.8	109.5	75.2	23.9	42.1	58.5	71.7	87.9	119.2
*Dietary fibre (g/day)*	15.4	7.5	5.8	9.7	13.9	19.6	29.4	13.9	6.1	5.7	9.3	12.9	17.8	24.4	14.6	6.8	5.8	9.5	13.3	18.7	27.0
*Dietary fibre (g/1000 kcal/day)*	8.4	2.6	4.8	6.6	8.2	9.9	13.4	9.2	2.9	5.2	7.1	8.9	11.0	14.6	8.8	2.8	5.0	6.9	8.5	10.4	13.9
**% Total energy from**																					
Protein (%En)	16	3	12	14	16	18	20	16	3	12	14	16	18	21	16	3	12	14	16	18	21
Fat (%En)	38	4	31	35	37	40	45	38	4	32	35	38	41	45	38	4	31	35	38	41	45
SFAs ^(a)^ (%En)	12	2	9	11	12	14	16	12	2	9	11	12	14	16	12	2	9	11	12	14	16
MUFAs ^(b)^ (%En)	17	3	13	15	17	19	22	18	3	14	16	17	19	22	17	3	13	16	17	19	22
PUFAs ^(c)^ (%En)	5	1	4	4	5	6	7	5	1	4	5	5	6	7	5	1	4	4	5	6	7
Carbohydrates (%En)	44	6	35	40	44	48	54	45	5	36	41	45	48	52	45	5	36	41	45	48	53
Total sugars (%En)	19	6	11	15	18	22	30	20	5	13	16	19	23	29	19	6	11	15	19	22	29
**Vitamins**																					
*Vitamin A (REs µg/day)*	787.8	319.6	383.4	581.2	741.7	936.7	1317	730.6	266.7	387.4	554.5	688.9	867.7	1180.3	758.3	293.9	384.5	570.7	711.8	900.3	1248
*β-carotene (µg/day)*	2915.9	1742	832.6	1685.7	2491.9	3839.6	6235.9	2842.6	1521.9	913.3	1749.6	2580.6	3609	5715.2	2878.3	1632.1	874.7	1723.9	2544.6	3703.8	5984.9
*Total folate (µg/day)*	258.3	112.4	111.2	174.4	237.7	327.1	456.2	230.0	95.5	109.1	160.4	214.3	283.4	398.7	248.6	104.9	114.9	172.6	229.7	311.7	431.5
*Dietary folate equivalent ^(e)^ (µg/day)*	263.4	112.0	118.3	180	243.7	331.8	458.4	234.7	96.1	113.7	164.7	219.3	288.4	402.8	243.8	104.5	111.2	167.5	224.6	306.1	428.2
*Vitamin B12 (µg/day)*	4.8	2.8	1.8	3.1	4.2	5.7	9.7	3.8	1.8	1.6	2.7	3.6	4.5	6.9	4.3	2.3	1.7	2.9	3.9	5.1	8.3
*Vitamin C (mg/day)*	104.2	56.6	36.7	65.7	94.1	132.2	209.1	98.2	50.4	35.1	62.3	87.1	123.9	199.7	101.1	53.4	36.2	63.7	90.7	127.1	203.2
*Vitamin D (µg/day)*	2.9	1.9	1.0	1.7	2.4	3.6	6.6	2.4	1.5	0.8	1.4	2.0	2.8	5.5	2.6	1.7	0.9	1.6	2.2	3.2	6.0
*Vitamin E (mg/day)*	11.5	4.9	4.7	8	10.7	14.4	20.7	10.2	3.7	4.9	7.4	9.7	12.5	16.8	10.8	4.4	4.7	7.7	10.2	13.5	19
**Minerals**																					
*Calcium (mg/day)*	774.7	232.9	427.9	614.4	756.7	911.5	1181.7	694.9	208.8	375.2	548	678.5	826.9	1055.8	733.6	224.1	399.9	575.6	716.6	864.8	1124.9
*Iron (mg/day)*	10.4	4.0	4.8	7.3	9.9	13.1	17.6	8.7	3.0	4.6	6.5	8.5	10.7	14.1	9.5	3.6	4.6	6.9	9.1	11.8	15.9
*Magnesium (mg/day)*	275.2	130.8	96.9	174.8	260.9	353.9	518.6	234.1	93.9	100.1	168.1	219.5	293	404.4	254.1	114.8	97.6	170.5	236.6	323.2	457.5
*Potassium (mg/day)*	2640.4	1087.4	1049.4	1792.5	2564.6	3363	4491.6	2341.4	835.5	1065.3	1738.9	2249.3	2908.6	3795.5	2485.9	975.9	1049.5	1757.1	2395.1	3110	4262.4
*Zinc (mg/day)*	10.0	3.8	4.8	7.2	9.5	12.2	17.1	8.3	2.5	4.8	6.6	8.1	9.7	13	9.1	3.3	4.7	6.8	8.7	11	15.2

^(a)^ SFAs: Saturated fatty acids; ^(b)^ MUFAs: mono-unsaturated fatty acids; ^(c)^ PUFAs: poly-unsaturated fatty acids; ^(d)^ SD: standard deviation; and ^(e)^ expresses the folate content of foods by considering the different bioavailability of naturally occurring folate and added folic acid.

**Table 5:** The usual intake of energy, macronutrients, and micronutrients from food and beverages in adults (18–64 years) by sex, compared with the Italian dietary reference values (DRVs).

	Males (*n* = 346)								Females (*n* = 380)							
	Mean	SD ^(d)^	P5	P25	P50	P75	P95	DRVs ^(e)^	Mean	SD ^(d)^	P5	P25	P50	P75	P95	DRVs ^(e)^
**Energy (kcal/day)**	2271.5	396.9	1633.2	2024.8	2242	2511.8	2951.9	2630 ^(f)^	1737	270.5	1325.6	1552.1	1715.9	1903.3	2197.8	2120 ^(f)^
**Energy (MJ/day)**	9.50	1.66	6.83	8.47	9.38	10.51	12.35	11.00	7.27	1.13	5.55	6.49	7.18	7.96	9.20	8.87
**Protein (g/day)**	93.8	18.7	64.5	82.2	92	104.2	125.2	63	70.8	11.9	51.3	62.3	70.3	78.4	92.3	54
*Protein (g/kg bw)*	1.2	0.3	0.7	1.0	1.2	1.3	1.7	0.9	1.1	0.3	0.8	0.9	1.1	1.3	1.6	0.9
*Animal protein (g/day)*	61	16.4	36.8	50	58.5	69.1	87.7		45.1	11.5	26	37.6	44.9	52.6	65.1	
*Animal protein (% on total)*	64.4	7.2	52.7	60.4	64.5	69.5	74.5		63.4	9.2	45.7	58.8	64.7	69.7	76.8	
**Fat (g/day)**	95	18.3	65.2	82.5	94.5	105.9	127.2		75.1	13.2	52.8	66.2	74.6	83.8	96.8	
*SFAs ^(a)^ (g/day)*	29.2	6.2	20	25	28.4	33.4	40.4		22.9	4.9	15.2	19.2	23.1	26.5	30.6	
*MUFAs ^(b)^ (g/day)*	44.3	9	29.9	38.4	43.7	50.4	58.9		35.5	6.8	24.9	30.6	34.9	40.1	47.9	
*PUFAs ^(c)^ (g/day)*	13.6	3.8	8	11	13.4	15.4	20.1		10.7	2.7	6.9	8.8	10.5	12.2	15.2	
*Cholesterol (mg/day)*	328.6	90.7	192.4	270.9	320.9	373.6	512.7		244.2	42.2	175.9	217.4	242.1	271.6	315.2	
**Carbohydrates (g/day)**	251.8	54.2	163.8	216.4	247.7	280.8	346.8		195.9	40.2	132.7	168.6	192.9	224.9	263.5	
*Starch (g/day)*	149.7	35.1	95.1	125.4	147.7	171.7	212.3		109.7	26.1	68.4	92.2	107.7	127.2	151.6	
*Total sugars (g/day)*	87.2	27.4	50.6	68.8	83.9	103.7	131.5		75.2	19.9	46.2	60.6	73.6	89	108.9	
*Dietary fibre (g/day)*	20.2	6.2	11.1	15.8	19.6	23.8	32.1	25 ^(h)^	17.2	5.1	10.1	13.2	16.9	20.5	25.7	25 ^(h)^
*Dietary fibre (g/1000 kcal/day)*	9.1	2.5	5.5	7.4	8.9	10.6	14.2	12.6–16.7 ^(l)^	10.2	2.4	6.7	8.5	10.1	11.8	14.3	12.6–16.7 ^(l)^
**% Total energy from**																
Protein (% En)	17	2	14	16	17	18	20	15	17	3	13	15	16	18	21	15
Fat (% En)	37	4	31	35	37	40	44	25–30 ^(g)^	39	2	35	37	38	40	43	25–30 ^(g)^
SFAs ^(a)^ (% En)	11	1	10	11	11	12	14	<10 ^(h)^	12	1	9	11	12	13	14	<10 ^(h)^
MUFAs ^(b)^ (% En)	18	3	14	16	17	19	22		18	2	15	17	18	20	22	
PUFAs ^(c)^ (% En)	5	1	4	5	5	6	7	5–10 ^(g)^	6	1	4	5	5	6	7	5–10 ^(g)^
Carbohydrates (% En)	42	5	35	39	42	45	50	45–60 ^(g)^	42	5	35	40	43	45	49	45–60 ^(g)^
Total sugars (% En)	16	4	10	13	16	18	22	<15 ^(h)^	18	4	12	15	17	20	23	<15 ^(h)^
**Vitamins**																
*Vitamin A (REs µg/day)*	940.8	310	529.1	739.6	898.5	1081.4	1397.6	750	832.2	280.7	452.8	655.2	783.5	968.2	1288.1	650
*β-carotene (µg/day)*	3772.8	1511.3	1743.3	2694.4	3540.9	4529.1	6617.4		3485.1	1526.3	1481.8	2410.8	3229.6	4268.2	6087.7	
*Total folate (µg/day)*	341.8	76.9	227.8	284.7	339	389.6	483.9	330	289.4	80.5	180.4	229.5	283.2	337.1	419.5	330 ^(i)^
*Dietary folate equivalent (µg/day)* ^(o)^	344.3	78.5	227.9	287.1	340.4	392.1	491.8		292.3	82.4	180.9	230.8	285.2	339.5	424.7	
*Vitamin B12 (µg/day)*	6.2	2.8	3.1	4.7	5.5	7.1	10.7	4 ^(l)^	4.6	1.2	2.9	3.8	4.5	5.3	6.7	4 ^(l)^
*Vitamin C (mg/day)*	129.3	50.8	63.2	92.8	121.2	153.8	225.9	105	120.4	50.5	52.4	81	113.8	150.2	213.8	85
*Vitamin D (µg/day)*	3.2	0.3	2.8	3	3.2	3.4	3.7	15	2.5	1	1.3	1.8	2.4	3	4.5	15
*Vitamin E (mg/day)*	14.5	3.9	8.8	11.7	14.3	16.8	21.5	13 ^(l)^	12.4	3.1	7.6	10.2	12.3	14.5	17.7	12 ^(l)^
**Minerals**																
*Calcium (mg/day)*	827.3	213.7	500.5	685.5	811	966.6	1205.5	950	731.4	218.6	413.8	574.5	706	871.1	1116.5	950 ^(m)^
*Iron (mg/day)*	13.2	2.5	9.5	11.4	13.2	14.7	17.6	10	10.4	2.2	7.1	8.8	10.3	11.8	14.2	18 ^(n)^
*Magnesium (mg/day)*	383.2	98	246.7	314.2	369.5	431.5	567.6	350 ^(l)^	299.6	74.6	193	245.1	292.4	344	431.7	350 ^(l)^
*Potassium (mg/day)*	3462.2	721.5	2291.7	2990.6	3404.5	3871.4	4755.5	4500 ^(h)^	2913.8	567.9	2007.8	2521.5	2864.7	3293.5	3953.9	4500 ^(h)^
*Zinc (mg/day)*	12.6	2.8	8.6	10.7	12.3	14.2	17.8	12	9.7	1.6	7.3	8.5	9.5	10.7	12.6	9

^(a)^ SFAs: Saturated fatty acids; ^(b)^ MUFAs: mono-unsaturated fatty acids; ^(c)^ PUFAs: poly-unsaturated fatty acids; ^(d)^ SD: standard deviation; ^(e)^ DVRs: Italian dietary reference values—values reported as PRI (population reference intake) unless otherwise indicated; ^(f)^ reference energy intake levels, considering average values for 18–29 years and 30–59 years for a PAL (physical activity level) of 1.6 and height: males, 1.7 metres, and females, 1.6 metres; ^(g)^ range of reference intake levels, expressed as RI (reference intake range for macronutrients); ^(h)^ reference intake levels, expressed as STD (suggested dietary target); dietary fibre STD: adults should consume at least 25 g of dietary fibre, even if the energy intake is <2000 Kcal/day; ^(i)^ the reference intake levels for folate for women of childbearing age and pregnant women do not include supplementation recommended for the prevention of neural tube defects; ^(l)^ reference intake levels reported as AI (adequate intake); ^(m)^ regardless of age, the PRI (population reference intake) is 950 mg/day in pre-menopausal women and 1100 mg/day in post-menopausal women; ^(n)^ the reference value for iron for women in menopause is 10 mg per day; and ^(o)^ expresses the folate content of foods by considering the different bioavailability of naturally occurring folate and added folic acid.

**Table 6:** The usual intake of energy, macronutrients, and micronutrients from food and beverages in elderly people (65–74 years) by sex, compared with the Italian dietary reference values (DRVs).

	Males (*n* = 65)								Females (*n* = 91)							
	Mean	SD ^(d)^	P5	P25	P50	P75	P95	DRVs ^(e)^	Mean	SD ^(d)^	P5	P25	P50	P75	P95	DRVs ^(e)^
**Energy (kcal/day)**	2188.6	533.9	1578.9	1787.8	2100.3	2458.8	3184.1	2160 ^(f)^	1659	348.1	1094.5	1399.1	1586.6	1926.9	2222.4	1890 ^(f)^
**Energy (MJ/day)**	9.16	2.23	6.61	7.48	8.79	10.29	13.32	9.04	6.94	1.46	4.58	5.85	6.64	8.06	9.30	7.91
**Protein (g/day)**	87.3	22.3	58.7	71.4	85	96.3	118.9	77 ^(g)^	67.2	14.2	46.5	57	66.5	77.2	89.9	66 ^(g)^
*Protein (g/kg bw)*	1.1	0.3	0.6	0.8	1.1	1.3	1.8	1.1 ^(g)^	1	0.3	0.7	0.8	1	1.1	1.5	1.1 ^(g)^
*Animal protein (g/day)*	55.0	16.4	31.4	42.7	52.1	65.1	82.7		41.8	11.4	25.8	33.2	41.5	49.4	62.3	
*Animal protein (% on total)*	61.6	7.7	48.1	57.8	62.8	66.4	72.6		61.4	6.6	50.2	57.1	61.8	66.1	71.1	
**Fat (g/day)**	87.9	23.2	57.9	72.2	80.5	98.7	133.4		68.4	15.4	46.8	58.3	66.8	79.2	96.4	
*SFAs ^(a)^ (g/day)*	25.5	7.2	15.5	20.8	24.4	29.5	37.8		21.4	6.2	13.5	16.4	21.2	25.8	31.5	
*MUFAs ^(b)^ (g/day)*	42.9	11.7	24.8	34.8	41.7	51.3	60.6		32.4	7.1	22.1	28	32	36.7	46	
*PUFAs ^(c)^ (g/day)*	12.2	4.4	7.4	9.1	11.8	13.7	19.9		9.1	2.8	5.8	7.2	8.5	10.6	15.1	
*Cholesterol (mg/day)*	296.6	88.1	183	227.8	292.1	357.1	447.9		220.4	46.6	156.8	184.6	215.8	253	300.8	
**Carbohydrates (g/day)**	240.3	62.5	156.3	194.1	233	277.8	368.1		194.3	44.6	121.4	163.1	194.7	226.2	275.1	
*Starch (g/day)*	141.2	43.4	75.9	111.2	135.4	168	221.8		105.9	31.1	61.1	82.2	103.2	129.7	168.4	
*Total sugars (g/day)*	84.9	24.1	47.2	68.4	86.8	100.4	123.6		76.7	15.6	52.9	67.4	75.3	85.5	99.8	
*Dietary fibre (g/day)*	23.5	7	13.2	18.6	23.1	26.2	37.6	25 ^(g)^	20.1	4.9	12.6	16.2	19.8	23.9	27.5	25 ^(g)^
*Dietary fibre (g/1000 kcal/day)*	11.2	2.1	7.6	9.9	11	12.2	15.4	12.6–16.7 ^(h)^	12.6	2.8	8.4	10.4	12.4	14.4	17.6	12.6–16.7 ^(h)^
**% Total energy from**																
Protein (% En)	16	2	13	15	16	17	19	15	16	2	14	15	16	18	20	15
Fat (% En)	36	4	29	33	35	39	42	20–30 ^(i)^	37	4	31	34	37	39	45	20–30 ^(i)^
SFAs ^(a)^ (% En)	10	2	7	10	11	12	13	<10 ^(g)^	11	2	9	10	11	13	15	<10 ^(g)^
MUFAs ^(b)^ (% En)	18	2	14	16	18	19	22		18	3	14	16	18	20	23	
PUFAs ^(c)^ (% En)	5	1	4	4	5	5	6	5–10 ^(i)^	5	1	3	4	5	5	7	5–10 ^(i)^
Carbohydrates (% En)	42	5	34	38	42	45	49	45–60 ^(i)^	44	5	37	41	45	47	51	45–60 ^(i)^
Total sugars (% En)	16	4	11	13	16	19	22	<15 ^(g)^	19	2	16	18	19	20	22	<15 ^(g)^
**Vitamins**																
*Vitamin A (REs µg/day)*	1165.5	325.3	609.1	1019.5	1157.2	1347.2	1512.3	750	920.1	234.3	540.3	787.3	918.4	1046.9	1247.5	650
*β-carotene (µg/day)*	4334	1317.6	2314.1	3331.3	4460.8	5352.7	6618		4038.1	586.2	2949.7	3653	4044.3	4417.1	4945.1	
*Total folate (µg/day)*	335.4	95.7	199	261.6	325.5	389.5	509.6	330	299.8	75.3	177.4	250.9	289.1	353.5	417	330
*Dietary folate equivalent (µg/day)* ^(m)^	335.5	95.9	198.9	261.5	325.5	389.5	509.7		301.7	76.4	177.4	251.4	289.9	354	417.9	
*Vitamin B12 (µg/day)*	7.2	1.8	5	6.1	7	8	10.4	4 ^(h)^	4.3	1.5	2.2	3.3	4.2	4.8	7	4 ^(h)^
*Vitamin C (mg/day)*	163.8	85.4	59.6	96.1	157.2	211.1	292.4	105	138.4	54.1	74.7	93.3	129.6	174.2	246.4	85
*Vitamin D (µg/day)*	2.6	0.1	2.3	2.5	2.6	2.6	2.7	15	2	0.6	1.1	1.7	2.1	2.3	2.9	15
*Vitamin E (mg/day)*	15.1	4.2	8.7	12.5	14.8	18	21.8	13 ^(h)^	11.8	2.2	8.2	10.3	11.9	13.1	15.5	12 ^(h)^
**Minerals**																
*Calcium (mg/day)*	818	238.6	511.9	649.3	805.2	916.1	1332.6	1100	778.2	174.6	467.5	664.4	809.5	885.7	1062.1	1100 ^(l)^
*Iron (mg/day)*	14.3	3.2	9.8	12.4	13.8	16	19.8	10	10.4	2	7	9	10.6	11.9	13.6	10
*Magnesium (mg/day)*	365.4	112.2	224.1	285.6	346.7	428.8	557.4	350 ^(h)^	291.4	61.8	199.6	243.4	285.9	337.2	397	350 ^(h)^
*Potassium (mg/day)*	3616.9	890.1	2235.5	3047.1	3547.6	4055.5	5140.8	3900 ^(g)^	3011.3	420.8	2299.4	2755.4	3007.8	3245.9	3820.6	3900 ^(g)^
*Zinc (mg/day)*	12.3	2.5	8.6	10.4	11.9	14.2	16.9	12	9.4	1.2	7.3	8.5	9.4	10.3	11.3	9

^(a)^ SFAs: Saturated fatty acids; ^(b)^ MUFAs: mono-unsaturated fatty acids; ^(c)^ PUFAs: poly-unsaturated fatty acids; ^(d)^ SD: standard deviation; ^(e)^ DVRs: Italian dietary reference values—values reported as PRI (population reference intake) unless otherwise indicated; ^(f)^ reference energy intake levels considering values for 60–80 years, a PAL (physical activity level) of 1.6 and height: males, 1.7 m, and females, 1.6 m; ^(g)^ reference intake levels reported as STD (suggested dietary target); dietary fibre STD: adults should consume at least 25 g of dietary fibre, even if the energy intake is <2000 Kcal/day; ^(h)^ reference intake levels reported as AI (adequate intake); ^(i)^ range of reference intake levels, expressed as RI (reference intake range for macronutrients); ^(l)^ regardless of age, the PRI (population reference intake) is 950 mg/day in pre-menopausal women and 1100 mg/day in post-menopausal women; and ^(m)^ expresses the folate content of foods by considering the different bioavailability of naturally occurring folate and added folic acid.

**Table 7:** Percentage contribution of food categories to total intake of energy, macronutrients, and fibre for total sample (*n* = 1969).

Food Groups	Energy	Protein	Animal Protein	Plant Protein	Fat	SFAs ^(a)^	MUFAs ^(b)^	PUFAs ^(c)^	Cholesterol	Carbohydrates	Total Sugars	Dietary Fibre
	%	%	%	%	%	%	%	%	%	%	%	%
Cereals, cereal products and substitutes	38.8	25.9	2.8	69.8	18.6	19.4	14.5	25.1	22.1	62.5	22.7	42.3
Pulses	0.8	1.6	0.0	4.6	0.2	0.1	0.1	0.5	0.0	1.1	0.4	5.0
Vegetables, fresh and processed	1.9	3.4	0.0	9.9	0.7	0.4	0.2	2.2	0.0	2.4	5.8	19.1
Potatoes, tubers, and their products	2.2	1.2	0.0	3.5	1.2	1.0	0.5	4.0	0.0	3.5	0.2	4.3
Fruit, fresh and processed	5.8	2.5	0.0	7.3	3.3	1.2	3.1	8.0	0.0	9.3	21.8	23.8
Meat, meat products, and substitutes	10.0	30.9	47.0	0.3	13.0	14.8	11.4	14.0	28.1	0.3	0.4	0.3
Fish, seafood, and their products	2.3	8.6	13.1	0.1	2.0	1.3	1.3	4.5	8.1	0.3	0.3	0.0
Milk, milk products, and substitutes	14.9	21.2	32.0	0.6	21.1	35.4	13	8.1	18.1	7.9	18.6	1.8
Oils and fats	12.9	0.1	0.1	0.0	33.7	18.4	51.1	28.4	1.6	0.0	0.1	0.0
Eggs	1.2	2.5	3.8	0.0	2.0	2.0	1.7	2.0	20.1	0.0	0.0	0.0
Sweet products and substitutes	4.7	1.2	1.0	1.5	3.7	5.6	2.8	2.1	1.7	7.2	16.6	2.2
Water and other non-alcoholic beverages	2.3	0.7	0.1	2.0	0.1	0.1	0.1	0.2	0.0	4.8	11.5	0.9
Alcoholic beverages and substitutes	2.0	0.1	0.0	0.2	0.0	0.0	0.0	0.0	0.0	0.6	1.6	0.0
Meal substitutes	0.0	0.1	0.1	0.1	0.0	0.0	0.0	0.0	0.0	0.0	0	0.1
Miscellaneous	0.2	0.1	0.0	0.3	0.4	0.3	0.3	0.9	0.1	0.1	0.1	0.1

^(a)^ SFAs: Saturated fatty acids; ^(b)^ MUFAs: mono-unsaturated fatty acids; and ^(c)^ PUFAs: poly-unsaturated fatty acids.

**Table 8:** Percentage contribution of food categories to total intake of minerals and vitamins for total sample (*n* = 1969).

Food Groups	Ca	Fe	Mg	K	Zn	Vit A Eq	β-Carotene	Folate Eq	Vit B12	Vit C	Vit D	Vit E
	%					%						
Cereals, cereal products, and substitutes	11.4	34.2	25.7	14.1	22.0	6.4	1.7	28.5	5.2	2.3	13.5	12.6
Pulses	0.6	3.6	3.2	2.0	1.3	0.3	0.5	5.4	0.0	1.3	0.0	0.3
Vegetables, fresh and processed	7.1	11.9	9.7	16.1	8.6	43.4	69.7	24.6	0.1	37.6	0.8	11.4
Potatoes, tubers, and their products	0.5	2.3	3.9	8.3	4.5	0.1	0.2	5.1	0.0	5.2	0.0	1.1
Fruit, fresh and processed	4.3	8.3	9.8	16.1	3.5	11.9	19.1	9.7	0.5	31.7	0.1	10.1
Meat, meat products, and substitutes	1.6	14.9	9.0	12.8	24.3	6.4	0.7	4.6	31.7	0.3	17.1	1.6
Fish, seafood, and their products	1.9	5.1	3.5	4.0	5.4	1.2	0.0	1.1	27.6	0.2	32.3	3.8
Milk, milk products, and substitutes	54.0	6.9	12.2	13.1	23.3	20.0	2.2	12.7	29.0	7.4	25.4	7.1
Oils and Fats	0.2	0.4	0.0	0.0	0.1	1.5	0.3	0.0	0.1	0.0	0.7	46.2
Eggs	0.9	2.1	0.7	0.7	1.7	3.7	0.4	2.7	3.9	0.0	8.8	1.9
Sweet products and substitutes	2.6	3.4	2.6	2.1	1.6	1.8	0.3	0.7	1.1	0.7	0.9	0.7
Water and other non-alcoholic beverages	14.3	3.5	16.8	9.0	3.2	2.9	4.6	3.8	0.4	13.1	0.3	1.8
Alcoholic beverages and substitutes	0.3	3.0	2.3	1.0	0.0	0.0	0.0	0.5	0.1	0.2	0.0	0.0
Meal substitutes	0.1	0.1	0.1	0.0	0.0	0.0	0.0	0.0	0.0	0.0	0.1	0.0
Miscellaneous	0.2	0.2	0.2	0.7	0.2	0.2	0.2	0.4	0.1	0.0	0.0	1.1

## Data Availability

Statistics on chronic and acute food consumption are available on the EFSA webpage: https://www.efsa.europa.eu/en/data-report/food-consumption-data (accessed on 27 October 2025).

## Supplementary Materials

The following supporting information can be downloaded at: https://www.mdpi.com/article/10.3390/nu18010088/s1. Tables S1–S7: Nutrient intakes, Table S1: Usual intakes of energy, macronutrients, and micronutrients in total population (3 month–74 years) by sex—from food and beverage sources only, all nutrients and percentiles; Table S2: Usual intakes of energy, macronutrients, and micronutrients in infants (3–11 months) by sex—from food and beverage sources only, all nutrients and percentiles; Table S3: Usual intakes of energy, macronutrients, and micronutrients in toddlers (1–2 years) by sex—from food and beverage sources only, all nutrients and percentiles; Table S4: Usual intakes of energy, macronutrients, and micronutrients in children (3–9 years) by sex- from food and beverage sources only, all nutrients and percentiles; Table S5: Usual intakes of energy, macronutrients, and micronutrients in adolescents (10–17 years) by sex—from food and beverage sources only, all nutrients and percentiles; Table S6: Usual intakes of energy, macronutrients, and micronutrients in adults (18–64 years) by sex—from food and beverage sources only, all nutrients and percentiles; and Table S7: Usual intakes of energy, macronutrients, and micronutrients in elderly people (65–74 years) by sex—from food and beverage sources only, all nutrients and percentiles. Tables S8–S14: Dietary sources, Table S8: Percentage contribution of food categories to total intake of energy, macronutrients, and micronutrients in infants (3–11 months); Table S9: Percentage contribution of food categories to total intake of energy, macronutrients, and micronutrients in toddlers (1–2 years); Table S10: Percentage contribution of food categories to total intake of energy, macronutrients, and micronutrients in children (3–9 years); Table S11: Percentage contribution of food categories to total intake of energy, macronutrients, and micronutrients in adolescents (10–17 years); Table S12: Percentage contribution of food categories to total intake of energy, macronutrients, and micronutrients in adults (18–64 years); Table S13: Percentage contribution of food categories to total intake of energy, macronutrients, and micronutrients in elderly people (65–74 years); and Table S14: Percentage contribution of food categories to total intake of energy, macronutrients, and micronutrients in total population (3 months–74 years).

## Abbreviations

The following abbreviations are used in this manuscript:

AI	Adequate Intake
AR	Average Requirement
BMI	Body Mass Index
BMR	Basal Metabolic Rate
Ca	Calcium
CHO	Carbohydrates
CREA	Consiglio Per La Ricerca In Agricoltura E L’analisi Dell’economia Agraria
DFE	Dietary Folate Equivalents
DRVs	Dietary Reference Values
EFSA	European Food Safety Authority
En	Energy Intake
Fe	Iron
INRAN-SCAI	Third Italian National Food Consumption Survey
IV SCAI	Fourth Italian National Food Consumption Survey
K	Potassium
Mg	Magnesium
MJ	Megajoule
MSM	Multiple Source Method
MUFA	Monounsaturated Fatty Acids
NCDs	Non-Communicable Diseases
P	Phosphorus
PAL	Physical Activity Level
PRI	Population Reference Intake
PUFA	Polyunsaturated Fatty Acids
RI	Reference Intake Range for macronutrients
SDT	Suggested Dietary Target
SFA	Saturated Fatty Acids
WHO	World Health Organization
Zn	Zinc

## Appendix A

Each professional who has completed the training courses has become a highly qualified specialist in the harmonised European-level detection methods for the paediatric and adult populations.

The members of the IV SCAI fieldwork team, listed below, formed this important community of highly specialised fieldworkers who made it possible to carry out the Italian national survey on food consumption.

**Table A1:** Members of the IV SCAI fieldwork team.

Nicole Acquaviva	Maria Albini
Rosaria Amabile	Enrico Apreda
Alice Baganha Sabatino	Cristina Baggio
Eliane Denise Bahbouth	Rossella Ballotta
Francesca Barberi	Sandra Bassini
Lauretta Bianco	Lucia Bonadies
Francesca Borghi	Giuseppa Bruno
Filomena Capasso	Sabrina Capineri
Rosa Carbone	Roberta Carli
Liliana Cassano	Vincenza Castiglia
Maurizio Cavallaro	Ketty Ceccarelli
Sara Ciacci	Rosalba Cipresso
Lisa Colladet	Stefania Corradi
Anna Maria Covarino	Alessandra Covino
Maria Cristina Cucugliato	Marta D’Ambrosio
Sara Dattoli	Maria De Marinis
Ignazio Dei	Federica Del Genio
Giulia Dellacostanza	Maria Assunta Di Cesare
Flora Di Tommaso	Emanuela Alessandra Donghi
Roberta Falcone	Federica Falvo
Elena Felloni	Anna Ferrante
Antonella Ferrigno	Antonella Foglia
Paola Golzio	Silvana Grasso
Daniele Grumiro	Emilia Guberti
Marina Infante	Gianna Lia Innocenti
Domenica Iofrida	Marina La Rocca
Valeria Laganà	Patrizia Lamberti
Elisa Lazzarino	Carmela Legorano
Silvia Lisciani	Teresa Loiacono
Rosanna Macaluso	Manuela Maione
Monica Maj	Lara Marangoni
Cecilia Mari	Valeria Marmo
Domenico Marotta	Piero Maruccia
Nicola Marulla	Marika Massaro
Guido Antonello Mattera Ricigliano	Maria Rita Milone
Antonio Molinaro	Erika Mollo
Mariantonia Monni	Laura Morisi
Patrizia Orsini	Giuseppa Pacella
Michele Parmato	Laura Parravicini
Angela Pasinato	Brunella Pasquini
Franca Pasticci	Emma Petrella
Alessandro Pinto	Clorinda Pittalà
Angelica Pizzolante	Carolina Poli
Luigi Polverino	Lucia Pomaro
Paola Pozzo	Antonio Pratesi
Angela Pugliese	Sofia Pugnaloni
Marina Putzolu	Sara Quattrini
Lisa Randisi	Valeria Rebonato
Vittoria Rocchino	Paola Rossi
Gaia Rovai	Monica Ruotolo
Daniela Russo	Carmen Santangelo
Sara Santilli	Agata Rita Santonocito
Sabrina Scelfo	Egeria Scoditti
Angela Silvestri	Gabriella Siniscalchi
Giuseppa Smeralda	Vincenzo Sofia
Silvia Soligon	Erminia Solomita
Ottavia Sorace	Angela Spadafranca
Susanna Tardonato	Paola Tei
Paride Travaglini	Francesca Trinchella
Maria Trippa	Silvia Tulone
Salvatore Vaccaro	Anna Valente
Viviana Vecchio	Lorella Vicari
Sara Vignozzi	Laura Zangari

## Appendix B

**Table A2:** Food items included in food groups: Italian National Food Consumption Survey IV SCAI.

Food Groups	Single Food Items and Composite Food Items Merged into	Minor Ingredients from Other Food Groups Present in Composite Foods	Subgroups Merged into
Cereals, cereal products and substitutes			
*Bread*	All types of bread prepared with any type of flour (wheat, whole wheat, soya, maize, etc.), speciality breads, sponge bread, excl. breadcrumbs and toasted bread	Milk and fats in speciality breads; soya in soya bread	
*Pasta & pasta substitutes*	All types of pasta, incl. rice noodles, fresh pasta with eggs, tortellini and ravioli (fresh pasta with meat filling), puff pastry dough; Pasta for infants, also fortified	Meat, vegetables, cheese in fresh pasta filling; eggs in fresh pasta; butter in puff pastry dough	Including *Infant food–pasta*
*Pizza*	Plain white pizza, plain tomato pizza, and pizza doughs	Tomato in plain tomato pizza; oil in plain white and tomato pizza	
*Rice*	All types of rice, white and brown, excl. rice in commercial rice pudding		
*Grains and flour*	Wheat flour and whole wheat flour used in recipes, wheat germ, couscous, breadcrumbs; All types of flour (corn, oat, millet, barley, rice, etc.) used in recipes; Instant cereal-based preparations, cereal-based baby foods, also fortified	Fruit in some cereal-based baby foods	Including *Wheat flours; Other cereals & flours, Infant food–other cereals*
*Breakfast cereals*	All types of ready-to-eat cereals (generally used at breakfast with milk): corn flakes, puffed wheat, dried and toasted rice, muesli, etc.	Dried fruit and nuts in muesli; sugar; cocoa	
*Biscuits*	All types of sweet biscuits, incl. sugar-free biscuits, gluten-free biscuits; Biscuits for infants, granulated biscuits, also fortified	Sugar; eggs; fats; cocoa; dried fruit and nuts;	Including *Infant food–biscuits*
*Savoury fine bakery products*	All types of crackers, breadsticks, rusk, popcorn, salty appetizers and savoury biscuits	Fats, tomatoes, cheese, seeds, etc.	
*Cakes and sweet snacks*	Plain cakes without chocolate and chocolate coatings, without cream and custard and jam, etc., sweet cereal- based snacks (e.g., bars); Sweet rolls, all types of cakes with chocolate and chocolate coatings, with fruit, with cream, etc	Fats; eggs; fruit; milk; cocoa; cream	Including *Cakes and sweet snacks without and with creams*
**Pulses**			
*Pulses, fresh and processed, dried*	All types of pulses fresh and processed: lentils, peas, chickpeas, soyabeans, all types of beans excl. green beans; All types of pulses dried and processed: lentils, peas, chickpeas, soyabeans, all types of beans excl. green beans; Legume-based baby foods, powdered preparations based on legumes		Including *Pulses, dried; Infant food–pulses*
**Vegetables, fresh and processed**			
*Leafy vegetables, fresh*	Chicory, lettuce, spinach, etc.		
*Tomatoes, fresh*	Fresh tomatoes used in salad and other recipes (e.g., tomato sauce)		
*Other fruiting vegetables, fresh*	Aubergine, pepper, cucumber, pumpkin, courgette, etc.		
*Roots and onions, fresh*	Onion, garlic, turnip-rooted celery, beetroot, daikon		
*Other vegetables, fresh*	All other types of fresh vegetables: broccoli, cabbage, cauliflower, artichokes, asparagus, fennel, mushroom, green beans and palm heart		
*Vegetables, processed*	Tomato sauce canned, bottled etc., tomato paste; Vegetable preserves in vinegar, oil or brine; Vegetable soup dehydrated, dried seaweed; Industrial mixed salad with mayonnaise, mixed vegetable preparations for rice salad, vegetable broth; Vegetables-based baby foods	Oil (in oil preserves); vinegar (in oil and vinegar preserves); Cereals and potatoes (in dehydrated soup); Eggs (in mayonnaise)	Including *Tomatoes, canned; Other vegetables, canned; Vegetables, dried; Other vegetables, packaged products; Infant food–vegetables*
*Spices and herbs*	Parsley, basil, rosemary, oregano, curry, ginseng, etc.		
**Potatoes, tubers and their products**			
*Potatoes and potato products, excl. potato chips*	Potatoes raw, sweet potatoes, potato croquettes, potato flakes dehydrated; Tapioca, yam root	Cheese in some speciality potato; breading in potato croquettes	Including *Other tubers*
*Potato chips and french fries*	All potato chips, frozen french fries	Fats in all potato chips; cheese in some speciality potato chips	
**Fruit, fresh and processed**			
*Citrus fruit, fresh*	Orange, lemon, grapefruit, tangerine, etc.		
*Berry fruit, fresch*	Strawberries, raspberries, blueberries, blackberries, goji berries, etc		
*Exotic fruit, fresh*	Banana, cherimoya, avocado, papaya, mango, lychee, etc		
*Other fruit, fresh*	All other types of fruit: apple, pear, peach, apricot, grape, fig, melon, watermelon, pomegranate, plum, cherry, etc.; Fruit-based baby foods		Including *Infant food–fruit*
*Nuts, dried fruit, seeds, olives and their products*	Nuts roasted, dried, in powder or in purée (almond, chestnut, walnut, coconut, pine nut, peanut, pistachio), seeds (pumpkin seed) and all dried fruit (figs, plum, raisin), olives		
*Processed fruit* *(in syrup, in purée,* etc.)	All types of fruit in syrup (peach, apricot, pear, fruit cocktail. etc.), fruit purée, minced fruit	Sugar	
**Meat, meat products and substitutes**			
*Beef & veal, not preserved, excl. offal*	Beef, veal, industrial meat sauce	Tomato in industrial meat sauce	
*Pork, not preserved, excl. offal*	Pork meat, roasted pork meat (porchetta), foot pork raw, excl. offal		
*Poultry and game, not preserved, excl. offal*	Pheasant, chicken, roast chicken, goose, quail, turkey, ostrich		
*Other meats, not preserved, excl. offal*	Lamb, goat, mutton, kid, horse, donkey, lean deer, boar, rabbit, frog, land snail, excl. offal		
*Ham, salami, sausages, excl. offal*	Preserved meat from pork, chicken and turkey meat (ham, mortadella, sausages, wurstel, salami, etc.), dried beef or horse meat (bresaola); Corned beef, mixed wurstel stuffed with cheese or other ingredients; Meat-based baby foods	Cheese; Fats, cereals and tubers in meat-based baby foods	Including *Other meats, preserved, excl. offal; Infant food_meat*
*Offal, and their products*	Liver, kidney, brain, trotter, heart, tripe, tongue, sweetbreads, blood and liver pate’	Fats in liver pate’	
*Meat substitutes*	Seitan (wheat gluten), soya hamburger	Soya; wheat	
**Fish, seafood and their products**			
*Fish and seafood, fresh and frozen*	All types of fish raw (fresh or frozen) and fish fingers; All types of molluscs (clams, oysters, mussels, octopus, squid, cuttlefish, etc.) crustaceans (shrimps, prawns, crabs, lobsters, crayfish, etc.), raw (fresh or frozen)	Breading on fish fingers	Including *Seafood, fresh and frozen*
*Fish and seafood, preserved*	All types of preserved fish, molluscs, crustaceans and fish eggs (caviar, anchovies brined or in oil, tuna brined or in oil, smoked salmon, canned crab meat, cod dried and salted, smoked herring, etc.); Fish-based baby foods	Oil in oil preserves; lemon; Fats, cereals, vegetables and tubers in fish-based baby foods	Including *Infant food–fish*
**Milk, milk products and substitutes**			
*Milk and milk-based beverages and substitutes*	All types of milk (liquid, condensed and powder form), flavoured milk (e.g., packaged chocolate-flavoured milk); Milk substitutes (e.g., soya, rice, oat); Breast milk; Ready-to-feed liquid formula (growing up milk, follow- on milk) for infants; Powdered milk formulas (growing-up milk and follow- on milk) for infants	Sugar in flavoured milk beverages; cocoa in flavoured milk beverages	Including *Milk substitutes plant-based; Human milk; Infant formula, liquid; Infant formula, powder*
*Yoghurt and fermented milk*	All types of yoghurt, drinkable yoghurt, milk with ferments (e.g., kefir); Yoghurt-based dessert with fruit for infants	Sugar in yoghurt; fruit, cereals, chocolate or nuts in yoghurt; fruit	Including *Infant food–yoghurt*
*Cheese and substitutes*	All types of cheese (e.g., mozzarella, parmesan, edam, feta, fontina), cheese substitutes (e.g., tofu); Flavoured sweet cheese for children, cheese-based baby foods	Fruit or vegetables and sugar in flavoured cheese for children	Including *Infant food–cheese*
			
*Milk-based desserts and substitutes*	Commercial milk-based desserts (e.g., mousse chocolate, custard, Chantilly custard), incl. commercial creamy rice pudding (risolatte)	Sugar; milk cream; cocoa; rice in rice pudding	
**Oils and fats**			
*Olive oil*	Extra-virgin olive oil, olive oil, incl. enriched type		
*Other vegetable oils*	All type of vegetable oils, excl. olive oil		
*Butter & creams*	Butter, cream, heavy cream		
*Other fats*	Lard, tallow; All types of margarine, incl. soyabean butter; Mayonnaise, included light type	Eggs in mayonnaise	Including *Margarine; Mayonnaise and fat-based sauces*
**Eggs**	All types of eggs (chicken, duck, ostrich, etc.) excl. fish eggs		
**Sweet products and substitutes**			
*Ice cream, ice lolly and substitutes*	All types of ice creams (with all possible flavours and ingredients), ice lolly	Milk in ice cream; soya in soya ice cream; fruit; eggs	
*Chocolate and substitutes*	All types of chocolate (milk, white, cocoa 70–90%, with hazelnuts, etc.), spreadable chocolate cream with or without hazelnuts, chocolate bars filled with mou	Milk in milk chocolate; nuts in some chocolate creams or bars; rice in some chocolate bars	
*Candies, jam and othersweet products (incl. sugar-free)*	All types of candies (e.g., mou, fudge, fondant, different flavours), chewing gum, jam, marmalade, nougat with almonds, glazed chestnuts (marrons glacés), peanut brittle, sesame brittle, incl. sugar-free products	Nuts; seeds	
*Sugar, fructose, honey*	Honey, sugar, fructose, maple syrup, royal jelly		
*Cocoa and cocoa-based powder*	Cocoa powder, mixed powder of cocoa with other ingredients, with or without sugar, with or without dehydrated milk	Sugar; milk	
*Artificial sweeteners*	All types of table-top products containing artificial sweeteners (e.g., aspartame, saccharin) in tablets, powder or liquid		
**Water and other non- alcoholic beverages**			
*Tap water*	Tap water (as such, in beverages or recipes)		
*Bottled water*	All types of commercial bottled water		
			
*Coffee, tea, and substitutes (incl. decaffeinated)*	All types of coffee (e.g., brewed, decaffeinated, espresso’), all types of tea (brewed, deteinated) with or without sugar, pearled barley coffee; Herbal tea in cup and ready to drink (e.g., chamomille)		Including *Herbal tea*
*Fruit and vegetable juices without artificial sweeteners*	All types of fruit and vegetable juices (e.g., nectar, carrot juice, orange juice), with or without the addition of water, sugar, incl. fortified products; All types of fruit juices, ready to drink and powdered herbal teas for infant		Including *Infant food_fruit juices and beverages (incl. powders)*
*Non-alcoholic beverages*	All types of carbonate beverages (e.g., cola, soda, ginger ale, orange, tonic water), energy drinks, sport drinks, syrups to be diluted, with sugar; All types of carbonate beverages (e.g., cola, soda, gingerale, orange, tonic water) sugar-free and with artificial sweeteners; All types of powdered and granulated drink preparations	Sugar in powders for the preparation of instant beverages	Including *Non–alcoholic beverages without artificial sweeteners; Non–alcoholic beverages with artificial sweeteners; Non–alcoholic beverages, powder*
**Alcoholic beverages and substitutes**			
*Regular wine and substitutes*	All types of wine (red, white), non-alcoholic’ wine, excl. sparkling wine		
*Beer, cider and substitutes*	Beer all types, non-alcoholic beer		
*Sweet wine, spumante, aperitif*	Sparkling wine, aperitif (campari, aperol, bagardi, etc.)		
*Spirits & liquors*	Fortified wine (e.g., porto, cherry, vermouth, dessert wine sweet or dry, appetizer), spirits (e.g., grappa, brandy, whiskey, rum), liquors (amaro, limoncello, etc.)		
**Meal substitutes**	Meal replacements in liquid form and bar form		
**Miscellaneous**			
*Non-fat-based sauces and condiments*	Vinegar, ketchup, mustard sauce, soya sauce, etc.; Bouillon cubes, sodium bicarbonate (for food use), isinglass, baking powder, meat extract; Liquid cake flavourings	Tomato and sugar in ketchup, milk, cereal, vegetables; Meat in meat bouillon cubes	Including *Broth cubes and other products; Flavors*
			
**Liquid foods**	All water and beverages, milk in liquid state, drinkable yoghurt		
**Solid foods**	All other food items incl. oil, dehydrated items (milk), powder for reconstitution of beverages, creamy desserts (yoghurt), sauces, etc.		
